# Potential Reduction of Symptoms With the Use of Persuasive Systems Design Features in Internet-Based Cognitive Behavioral Therapy Programs for Children and Adolescents With Anxiety: A Realist Synthesis

**DOI:** 10.2196/13807

**Published:** 2019-10-23

**Authors:** Ashley D Radomski, Lori Wozney, Patrick McGrath, Anna Huguet, Lisa Hartling, Michele P Dyson, Kathryn J Bennett, Amanda S Newton

**Affiliations:** 1 Department of Pediatrics University of Alberta Edmonton, AB Canada; 2 Centre for Research in Family Health IWK Health Centre Halifax, NS Canada; 3 Department of Psychology Dalhousie University Halifax, NS Canada; 4 Department of Pediatrics Dalhousie University Halifax, NS Canada; 5 Department of Psychiatry Dalhousie University Halifax, NS Canada; 6 Department of Community of Health and Epidemiology Dalhousie University Halifax, NS Canada; 7 Department of Health Research Methods, Evidence and Impact McMaster University Hamilton, ON Canada

**Keywords:** internet, cognitive behavioral therapy, computer-assisted therapy, persuasive communication, anxiety, children, adolescents, review, treatment effectiveness, clinical effectiveness, treatment efficacy, clinical

## Abstract

**Background:**

Internet-based cognitive behavioral therapy (iCBT) for children and adolescents is a persuasive system that combines 3 major components to therapy—therapeutic content, technological features, and interactions between the user and program—intended to reduce users’ anxiety symptoms. Several reviews report the effectiveness of iCBT; however, iCBT design and delivery components differ widely across programs, which raise important questions about how iCBT effects are produced and can be optimized.

**Objective:**

The objective of this study was to review and synthesize the iCBT literature using a realist approach with a persuasive systems perspective to (1) document the design and delivery components of iCBT and (2) generate hypotheses as to how these components may explain changes in anxiety symptoms after completing iCBT.

**Methods:**

A multi-strategy search identified published and gray literature on iCBT for child and adolescent anxiety up until June 2019. Documents that met our prespecified inclusion criteria were appraised for relevance and methodological rigor. Data extraction was guided by the persuasive systems design (PSD) model. The model describes 28 technological design features, organized into 4 categories that help users meet their health goals: primary task support, dialogue support, system credibility support, and social support. We generated initial hypotheses for how PSD (mechanisms) and program delivery (context of use) features were linked to symptom changes (outcomes) across iCBT programs using realist and meta-ethnographic techniques. These hypothesized context-mechanism-outcome configurations were refined during analysis using evidence from the literature to improve their explanatory value.

**Results:**

A total of 63 documents detailing 15 iCBT programs were included. A total of six iCBT programs were rated high for relevance, and most studies were of moderate-to-high methodological rigor. A total of 11 context-mechanism-outcome configurations (final hypotheses) were generated. Configurations primarily comprised PSD features from the primary task and dialogue support categories. Several key PSD features (eg, self-monitoring, simulation, social role, similarity, social learning, and rehearsal) were consistently reported in programs shown to reduce anxiety; many features were employed simultaneously, suggesting synergy when grouped. We also hypothesized the function of PSD features in generating iCBT impacts. Adjunct support was identified as an important aspect of context that may have complemented certain PSD features in reducing users’ anxiety.

**Conclusions:**

This synthesis generated context-mechanism-outcome configurations (hypotheses) about the potential function, combination, and impact of iCBT program components thought to support desired program effects. We suggest that, when delivered with adjunct support, PSD features may contribute to reduced anxiety for child and adolescent users. Formal testing of the 11 configurations is required to confirm their impact on anxiety-based outcomes. From this we encourage a systematic and deliberate approach to iCBT design and evaluation to increase the pool of evidence-based interventions available to prevent and treat children and adolescents with anxiety.

## Introduction

### Background

Anxiety is one of the most common and early emerging mental health concerns for children and adolescents [1], 20% of whom will experience an anxiety disorder in their lifetime [1]. Often presenting with a chronic and recurring course that extends into adulthood [2], anxiety disorders are associated with considerable developmental, psychosocial, and psychopathological impairments [1,3]. The effectiveness of cognitive behavioral therapy (CBT), an adaptive, skills-based psychotherapeutic approach, has been documented in numerous randomized controlled trials and is recommended as the first line treatment for children and adolescents with mild-to-moderate anxiety symptom severity [4-7]. Internet-based CBT (iCBT) aims to increase access and availability of this beneficial first line treatment [8,9] as the delivery of CBT content no longer hinges on face-to-face appointments with specialized therapists. Recent systematic reviews and meta-analyses have found iCBT to be comparably effective at reducing anxiety symptoms in children and adolescents relative to face-to-face CBT [8,10-12], and more effective in reducing symptoms than waitlist conditions [8,11,13-16]. Overall, these findings indicate that iCBT is an effective treatment option that can increase access to care.

iCBT is a complex intervention [17] and is not merely the upload of therapeutic material onto a Web page. iCBT programs incorporate 3 major components: (1) structured and standardized therapeutic content (ie, CBT), (2) technological features (ie, multimedia and email) used to deliver the content, and (3) interactions between the user and the program to engage users in iCBT content and features. These components are characteristics of a *persuasive system*—an intervention designed to change user’s attitudes and behavior toward their desired health goal [18-20].

### Motivation and Objective

To date, considerable variety exists in terms of how the 3 iCBT components have been incorporated into iCBT program design. Only 2 studies of iCBT effectiveness have attempted to identify or explain what program components can be used to optimize the therapeutic gains of users and for what reasons. Calear et al [21] explored whether the expertise of the adjunct support person had an effect on intervention outcomes (teacher-only support vs teacher plus mental health education officer support [21]), and Spence et al [22] tested for a difference in adolescents’ anxiety reductions because of the specificity of program content (a program with social anxiety disorder–specific content compared with generic anxiety disorder content [22]), but neither study reported a significant difference in outcomes. Therefore, an essential question that remains for the field is, “What components of iCBT work, for whom, and why?” Using realist synthesis methodology, we used a persuasive systems perspective to examine the following:

1. *What design and delivery components are described in studies of iCBT programs for children and adolescents with anxiety?*
2. *What are the components reported in studies that appear to explain the change in anxiety symptoms after completing iCBT?*

## Methods

### Study Design

The realist synthesis provided us with a mixed methods approach to generate proposed explanations (hypotheses) of how and why iCBT programs work despite variations in its design and delivery [23-25]. The synthesis was conducted using the steps recommended by Pawson and Tilley [25,26] and reported in accordance with the Realist and Meta-Narrative Evidence Synthesis: Evolving Standards II [27]. We set out to examine the technological features of iCBT (mechanisms), embedded within delivery and conditions or settings for use (program context), that produced changes in anxiety symptoms for children and adolescents (outcomes). Pawson and Tilley refer to these relationships as context-mechanism-outcome configurations. Thus, the overall purpose of the synthesis was to produce context-mechanism-outcome configurations that hypothesized when and how iCBT programs might be effective in reducing anxiety symptoms among children and adolescents with anxiety.

### Candidate Context-Mechanism-Outcome Configuration Development

We started the synthesis with the generation of a list of *candidate* context-mechanism-outcome configurations. We decided a priori to use an established, valid framework to guide the generation of candidate configurations. This also helped us maintain a consistent and streamlined approach to the synthesis (ie, extract and code data according to framework). We conducted an informal literature scan and held research team discussions to identify preexisting frameworks from the fields of psychology, pediatrics, human-computer interaction, and electronic health (eHealth). The persuasive systems design (PSD) model [28] was selected as the most appropriate framework to direct the candidate configurations and answer our research questions. It is a recent, well-studied model [29] that describes 28 technological design features that can be incorporated into a persuasive system to help the user meet his or her health goals (Multimedia Appendix 1 [30-43]). The model organizes the design features under 4 categories based on their main purpose: primary task support (assists the user in completing their target behavior), dialogue support (provides computer-human communication to guide user toward target behavior), system credibility support (increases user’s perceptions of a system’s credibility), and social support (leverages the interactions and influence of others).

We used the PSD model to identify PSD features (mechanisms) hypothesized to produce anxiety symptom changes (outcomes) in iCBT programs and recorded these as mechanism-outcome dyads. We then considered the program design and delivery features (context) that might support the operation of the mechanism-outcome dyads and combined them in unified but distinct configurations. The result was 8 candidate context-mechanism-outcome configurations (Multimedia Appendix 2), or initial hypotheses, that formed the basis of our analysis. We refined these configurations during the realist synthesis so that they reflected the iCBT literature. Following analysis, we considered our configurations to be fully developed hypotheses ready for future testing.

### Literature Search

We required diverse literature to inform this synthesis. We sought to include primary or secondary studies of iCBT interventions, conference proceedings, websites, program evaluations, and government or technical reports. We used 3 search strategies to identify this literature: (1) a systematic, comprehensive search of 8 electronic databases from disciplines relevant to the topic (ie, medicine and psychology)—Medline, Embase, Education Resources Information Center, PsycINFO, Cumulative Index to Nursing and Allied Health Literature, Cochrane Library, ProQuest Dissertations & Theses Global, and PubMed for the period from 1990 to 2017, conducted by an information specialist; (2) a manual search using an internet search engine (Google) and gray literature repositories (eg, Association for Computing Machinery Digital Library, Open Gray, Institute of Electrical and Electronics Engineers Digital Library, and Canadian Agency for Drugs and Technologies in Health); and (3) a hand search of medical informatics journals (*Journal of Medical Internet Research*, *Internet Interventions*, *Journal of Cybertherapy and Rehabilitation*, and *Journal of Telemedicine and Telecare*) and the reference lists of included documents and reviews (eg, systematic reviews). Medical Subject Headings terms and text words for the search were based on mental health condition (ie, anxiety and phobias), intervention modality (ie, internet-based and mobile apps), intervention type (ie, prevention and treatment), and therapeutic approach (ie, CBT; Multimedia Appendix 3). We applied the search strategies in an initial search (conducted up until February 2015) and then in 2 updated searches (conducted in November 2017 and in June 2019) to ensure the realist synthesis was current and inclusive.

### Document and Internet-Based Cognitive Behavioral Therapy Program Selection

We were interested in including documents relevant to iCBT programs that were designed for use by children or adolescents aged ≤19 years diagnosed with an anxiety disorder(s) or with anxiety symptoms associated with a disorder as classified according to the fifth edition of the Diagnostic and Statistical Manual of Mental Disorders [44]. Documents needed to include information about an iCBT program designed for treating child or adolescent anxiety and be available in English. Documents that detailed information for a transdiagnostic program (eg, treating an anxiety disorder plus depression) were eligible for inclusion but documents of programs designed solely for parent use were not. All eligible documents were grouped according to the iCBT program it represented (ie, program name). We included in the realist synthesis documents of programs that detailed its delivery context (ie, the conditions for program use), PSD mechanisms (ie, information on the technological features for how the program was proposed to work), and impact on anxiety outcomes after program delivery (ie, at least one published study of postintervention effects). These details could be described within 1 document, or across multiple documents, but needed to be available so that at least one context-mechanism-outcome configuration could potentially be generated for each iCBT program.

During the document selection progress, 2 reviewers (authors ADR and LW) independently applied the inclusion criteria using a 2-stage approach. In stage 1, titles and abstracts of documents were screened for potential eligibility (*yes*, *no*, or *unsure*). The reviewers randomly selected 100 documents to assess inter-rater agreement and found *substantial* agreement (Cohen kappa=0.74) [45]. In stage 2, the full text of *yes* or *unsure* documents were reviewed by the 2 reviewers for inclusion or exclusion in the synthesis. In both stages, they resolved discrepancies through discussion (with author ASN) until consensus was reached.

### Literature Appraisal

Quality appraisal of included documents involved assessing relevance to the synthesis objectives and, in the case of research studies, assessing methodological rigor. A total of 2 reviewers (authors ADR and LW) conducted the quality appraisal. Relevance was assessed by reviewing a document’s *level of contribution*, the extent to which information was provided on (1) theory and/or the context and sequences for iCBT delivery (context), (2) PSD features, required interactions by the deliverer/user and the program, and/or other proposed program mechanisms (mechanism), and (3) the impact of iCBT on anxiety symptoms outcomes and explanations for the findings (outcome). The level of contribution was rated *low* if little or no information was provided, *medium* if some information was provided, and *high* if information was well-described, relative to other documents for other programs.

To understand the quality of the research studies that provided outcome data for the synthesis, the methodological rigor of studies was assessed using the Mixed Methods Appraisal Tool (MMAT) [46,47]. The MMAT is a reliable, efficient, and validated tool that provides different sections for assessing studies of qualitative, randomized, nonrandomized, descriptive, and mixed methods design [46-48]. Multiple documents using the same, full dataset (ie, thesis plus published paper of the thesis) received the same MMAT score but was only counted once. MMAT scores could range from 0% to 100%, with a greater score signifying more rigorous criteria were met.

### Data Extraction and Coding

To identify context-mechanism-outcome configurations, we extracted and coded iCBT program data using a data matrix with 6 major domains: (1) document characteristics (eg, study design), (2) participant characteristics (eg, demographics) and study procedures (ie, eligibility criteria), (3) context of iCBT delivery including a program’s targeted level of prevention according to the Institute of Medicine (IOM) model [49] and adjunct support details (human-derived technological or therapeutic communication complementary to program delivery), (4) program theory and principles behind iCBT program design, (5) program components or proposed mechanisms (ie, CBT content and PSD features and interactions between the user and program), and (6) pre- to postintervention change in anxiety symptoms. For outcome data, not all studies reported within-group analyses; therefore, absolute changes in anxiety symptoms among children or adolescents who received iCBT were recorded. If original authors referred to previous publications of an iCBT program, we included the document and extracted relevant data. We also extracted partial or full context-mechanism-outcome configurations, if provided by the original authors.

We used the PSD model [20] and a customized codebook to guide the coding process (Multimedia Appendix 1). Similar to previous studies [29,50], PSD features of iCBT programs were coded: (1) when a program feature was executed by technology (eg, video demonstration of an adolescent performing deep breathing) rather than by human action (eg, a parent demonstrating deep breathing in person) and (2) when the feature was a part of the iCBT program, not supporting research study materials, such as an informational website. We coded therapeutic content according to the 5 main CBT components found in the American Academy of Child and Adolescent Psychiatry (AACAP) practice parameter for anxiety disorders [6]: psychoeducation, somatic management skills, cognitive restructuring, exposure methods, and relapse prevention. We also extracted other therapeutic content, such as behavioral activation details and interpersonal therapy techniques [51,52]. We contacted corresponding authors associated with each iCBT program to support accurate and complete extraction and coding of the data. Overall, 80% (12/15) of original authors associated with the included iCBT programs responded to the request for more information. The interpretation of data extraction and coding between 2 reviewers (authors ADR and LW) was checked with a random sample of 10 documents and achieved consensus before the remaining documents were coded by a single reviewer (author ADR).

### Analysis and Synthesis Process

Analysis was conducted at the program level [25], which meant that multiple documents or studies relating to each unique iCBT program were grouped together for analysis of the iCBT program as a whole. Programs were grouped according to their level of prevention using the IOM model [49]—whether they were a universal prevention, indicated prevention, or treatment program (*program type*). The IOM model recognizes that the target population (ie, children or adolescents with baseline anxiety symptoms and an associated level of risk for a disorder) differs according to program type and so does the conceptual focus of the intervention, and together, these differences may influence the fundamental design and delivery of a program (ie, context and clinical techniques) and the expected degree of change users may experience (ie, outcomes) [53].

**Figure 1:**
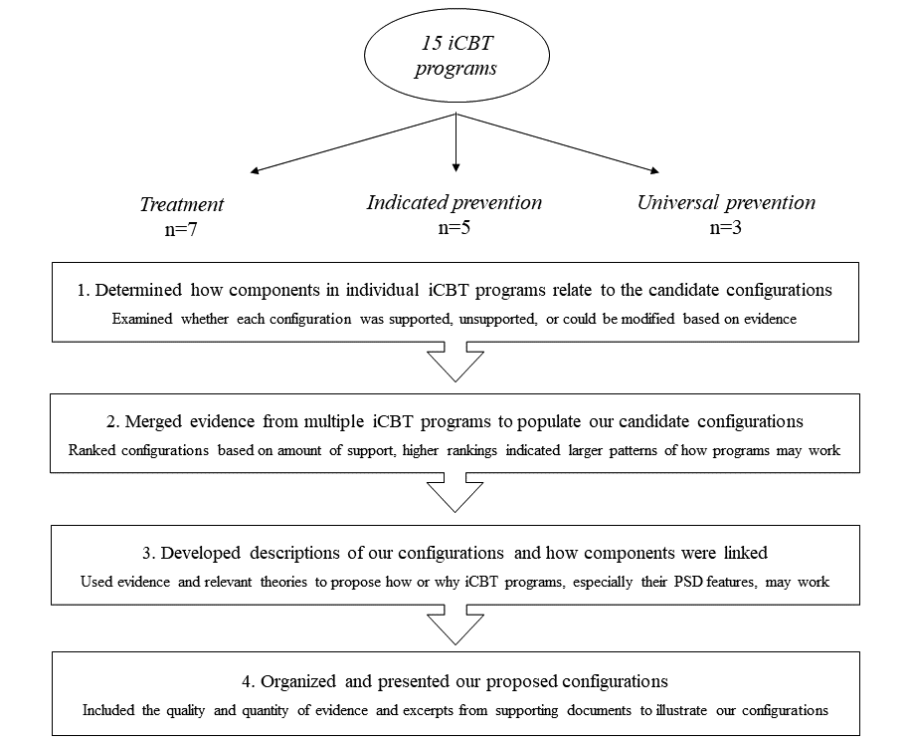
The 4 stages of the realist analysis and synthesis process of internet-based cognitive behavioral therapy (iCBT) programs for children and adolescents with anxiety. PSD: persuasive systems design.

We analyzed the candidate context-mechanism-outcome configurations in 4 stages using meta-ethnographic [54,55] and realist [24-26] techniques (Figure 1). During this process, information from included documents was synthesized to refine each configuration. In stage 1, we recorded the individual components (ie, contexts, mechanisms, and outcomes) and relationships between components (eg, mechanism-outcome dyads) reported in the documents for each iCBT program. We compared the information for each iCBT program with the candidate configurations (initial hypotheses) and documented whether a candidate configuration was supported, unsupported, modified, or newly generated based on the evidence. In stage 2, reciprocal translation analysis was used to determine if common contexts and mechanisms were being described across programs. The candidate configurations were ranked from the most to least supported, based on the number of iCBT programs supporting each configuration. A configuration was required to be supported by at least two iCBT programs to proceed with the next stage of analysis. We considered configurations with the highest rankings to depict the larger patterns or trends (demi-regularities) of iCBT program components. These candidate configurations became our final hypotheses for how iCBT programs were hypothesized to work.

In stage 3, we developed descriptions of how the iCBT program components were linked in our configurations. The descriptions focused on the proposed function (role) of key PSD features in explaining how iCBT programs might reduce anxiety for children and adolescents. To do this, we nested the configurations within our broader understanding of the theoretical underpinnings of the PSD model and CBT, along with original authors’ descriptions of the design or delivery of program features. This process allowed us to explore not only *what* iCBT program components might be working together but also *why* they might be doing so. We maintained a level of abstraction that allowed us to express the larger similarities across multiple programs while acknowledging the details that made each configuration unique (using lines-of-argument synthesis). This meant that we did not delve deeper into specific details of contexts, mechanisms, or outcomes. For example, we identified whether adjunct support was provided in programs rather than the specific amount of support provided, and we identified the direction of treatment effect rather than specific effect sizes. This approach was not only necessary because of the data available to us but also ensured that our configurations remained usable and applicable across the programs. During this stage, we also incorporated into the descriptions other factors that could help explain our understanding of the configurations (eg, multiple PSD features working together and differences in user characteristics). In stage 4, we identified the quantity (ie, number of programs) and quality (ie, relevance and methodological rigor) of support associated with each configuration. Research team meetings were used to discuss and improve the final detailed descriptions of our proposed configurations.

## Results

### Included Documents

**Figure 2:**
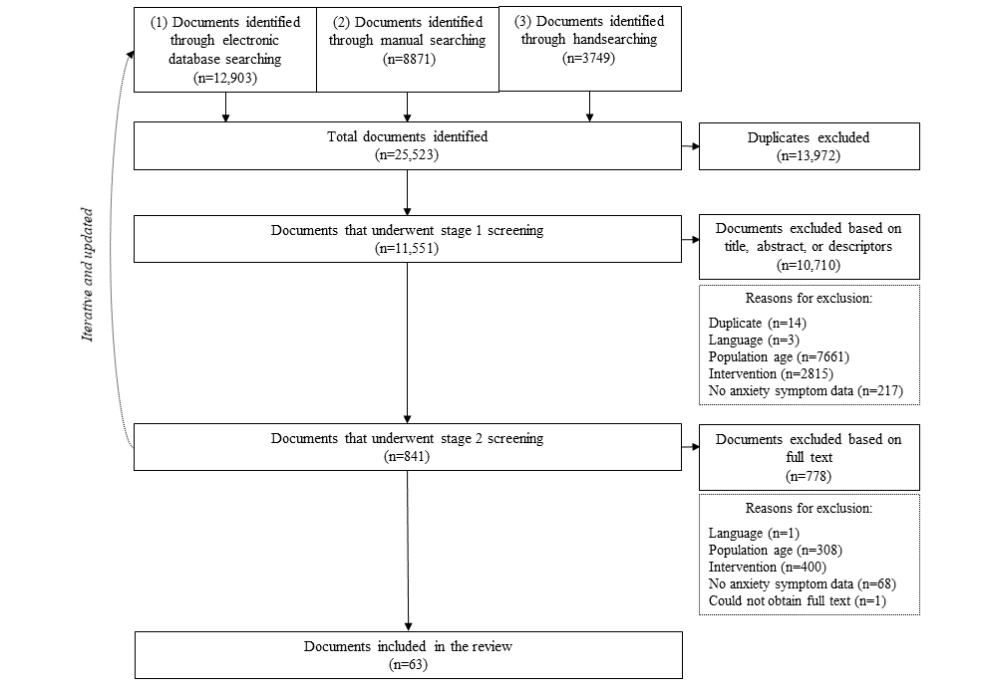
Flow diagram of the literature search and selection process.

Figure 2 is a flow diagram outlining the results of the 2-stage literature search and selection process. A total of 63 documents (30 from the initial search, 15 from the updated search conducted in November 2017, and 18 from the updated search conducted in June 2019) describing 15 iCBT programs were eligible and included in the review. The included documents were published studies (n=29), theses (n=5), registered protocols for trials (n=15), study or program websites (n=9), study flyers (n=2), and conference abstracts or posters (n=3).

### Level of Contribution and Methodological Quality

Details of the quality appraisal are provided in Multimedia Appendix 4 [21,22,30-39,41-43,56-91,92-102]. Across documents, the most details were provided for iCBT program contexts and outcomes. Program mechanisms were not so well described in terms of what the mechanisms were (ie, technological and therapeutic features) and how they were proposed to work. A total of 8 documents were rated as having a *high* level of contribution to understanding contexts, mechanisms, and outcomes [22,31,40,56-60]. These documents were for 6 iCBT programs that provided the greatest contributions to the context-mechanism-outcome configurations we developed: BRAVE-Online, internet-delivered CBT for children with anxiety disorders, internet-delivered CBT for children with specific phobia, internet cognitive behavioral skills–based program, STAY COOL system for test anxiety, and the e-couch Anxiety and Worry program.

MMAT scores were calculated for 35 research studies: 20 randomized controlled trials, 5 nonrandomized studies, 7 quantitative descriptive studies, 1 qualitative study, and 2 mixed methods studies. A total of 22 documents met all 4 MMAT criteria (100%), 7 documents met 3 criteria (75%), 5 documents met 2 criteria (50%), and 1 document met 1 criterion (25%). Lower MMAT ratings were a result of factors such as incomplete outcome data or unacceptable response rates, high withdrawal rates, or no mention of whether groups were comparable.

### Overview of the Contexts, Mechanisms, and Outcomes of Internet-Based Cognitive Behavioral Therapy Programs for Children and Adolescents With Anxiety

#### Contexts: User and Program Delivery Characteristics

Table 1 presents an overview of the user and program delivery characteristics of iCBT programs, organized according to program type. All programs were computer-based and included some form of adjunct support or program facilitation. Most programs (10/15 programs) were designed to solely target users with reported anxiety symptoms. There were similarities in the delivery setting, workflow, and adjunct support of iCBT programs of the same program type. Treatment programs were most often accessed at home, included 7 or more modules, and incorporated weekly Web-based therapist interaction and parent-dedicated modules. Indicated prevention programs demonstrated more variety in their use setting (eg, home, school, or clinic) and the adjunct support provided (eg, not all programs required parent participation), and the workflow tended to involve fewer modules (ie, typically 6 modules or less). Universal prevention programs were delivered with teacher facilitation in a classroom setting and incorporated the least number of modules relative to other program types. iCBT programs were based on relevant theoretical, anxiety, or CBT literature or published treatment recommendations [30,31,34,90,95], established clinic-based programs, manuals or workbooks [37,39,42,57,94], a school syllabus [35], or were adaptations of a developed iCBT program designed to target a different population or mental health condition [32,76,83,88].

**Table 1:** Overview of internet-based cognitive behavioral therapy user, program, and delivery characteristics.

Numbered list of programs^a^			Target users’ age group and symptom severity^b^			Program delivery						Therapist support in program										Adjunct program support	
						Use setting		# of sessions, frequency, or duration of program					Web or email				Phone	In-person					
**Treatment programs: 1, 2, 3, 4, 5, 6, 7**																							
	(1) BRAVE-Online			Children and adolescents with an anxiety disorder		Home					10 weekly sessions plus 2 booster sessions; 60 min each				X^c^	X				—^d^		Parent	
	(2) iCBT^e^ for dental anxiety			Children and adolescents with an anxiety disorder		Home plus clinic					12 weekly modules				X	—				—		Parent, dental professional^f^	
	(3) Internet-delivered CBT^g^ for children with anxiety disorders			Children with an anxiety disorder		Home					11 modules over a 10-week period				X	X				—		Parent	
	(4) Internet-delivered CBT for children with specific phobia			Children with an anxiety disorder		Home					11 modules over a 6-week period; 15-45 min each				X	X				—		Parent	
	(5) Chilled Out			Adolescents with an anxiety disorder		Home					8 modules over a 12- or 14-week period; 30 min each				—	X				—		Parent (optional)	
	(6) Group therapy supported iCBT for adolescents with social anxiety disorder			Adolescents with an anxiety disorder		Home plus clinic					12 weekly modules				X	X				X		Parent	
	(7) iCBT for anxiety disorders among adolescent girls			Adolescents with an anxiety disorder		Home					7 modules over a 3-month period; 1 hour daily				X	—				—		—	
**Indicated prevention programs: 8, 9, 10, 11, 12**																							
	(8) Internet cognitive behavioral skills-based program	Children with moderate-to-severe anxiety symptoms			Home				3 modules^h^ with 20 sections over a 12-week period					—		X			—			Parent	
	(9) Internet-supported brief CBT for shy-socially isolated problem	Adolescents with moderate-to-severe anxiety symptoms			School				6 modules					X		X			—			—	
	(10) STAY COOL system for test anxiety	Adolescents with mild-to-moderate anxiety symptoms			School or home				6 modules over 8 weeks; 20-30 min for each activity					—		—			X			Researcher^i^	
	(11) Feeling Better		Adolescents with mild-to-moderate anxiety and/or depressive symptoms		Home				4 modules^j^					X		X			—			—	
	(12) Individually tailored iCBT for adolescents		Adolescents with mild-to-severe anxiety and/or depressive symptoms		Clinic				6-9 prescribed modules over a 6- to 18-week period					X		X			X			—	
**Universal prevention programs: 13, 14, 15**																							
	(13) The e-couch Anxiety and Worry Program			Adolescents with no symptoms required			School			6 weekly sessions; 30-40 min each					—	—				—	Teacher^k^, mental health service provider^l^		
	(14) MoodGYM			Adolescents with no symptoms required			School			5 weekly modules; 30-60 min each					—	—				—	Teacher^k^		
	(15) Thiswayup Schools for Anxiety and Depression prevention courses			Adolescents with no symptoms required			School			6 (anxiety) or 7 (depression) weekly modules; 40 min each					—	—				—	Teacher^k^		

^a^Categorized according to the Level of Prevention Model [49]: universal prevention—target participants have not been identified on the basis of individual risk (ie, no symptoms required); selective prevention—target participants have a higher risk of developing an anxiety disorder than others; indicated prevention—target participants are at high risk, those who have anxiety signs or symptoms but do not currently meet diagnostic levels; and treatment—target participants are diagnosed with an anxiety disorder.

^b^Children: mean study age of users ≤12 years; adolescents: mean study age of users ≥13 years. The anxiety severity reported was the severity required for study inclusion; anxiety severity was not necessarily the baseline level of symptoms participants had.

^c^This type of therapist support was incorporated.

^d^This type of adjunct support was not incorporated.

^e^iCBT: internet-based cognitive behavioral therapy.

^f^A dental professional (a dentist, dental hygienist, or dental assistant) provided exposure at a dental clinic.

^g^CBT: cognitive behavioral therapy.

^h^2 blocks of modules (containing 9 major sections) are dedicated to mothers, and 1 module block (containing 12 major sections) is dedicated to the child plus mother.

^i^Research assistant or graduate student was present to facilitate aspects of the study, such as assessment and troubleshoot technical issues.

^j^The first 4 out of a possible 12 modules were delivered for the purpose of this study: Introduction, Activity and Motivation, Thoughts and Feelings, and Stress Management [95].

^k^Program administration was facilitated by a classroom teacher. The teacher was available for general guidance but did not provide an active therapeutic role in the program.

^l^A mental health service provider was present in 1 study of the program to facilitate program administration and address student questions [103].

#### Mechanisms: Therapeutic Content and Persuasive Systems Design Features

**Figure 3:**
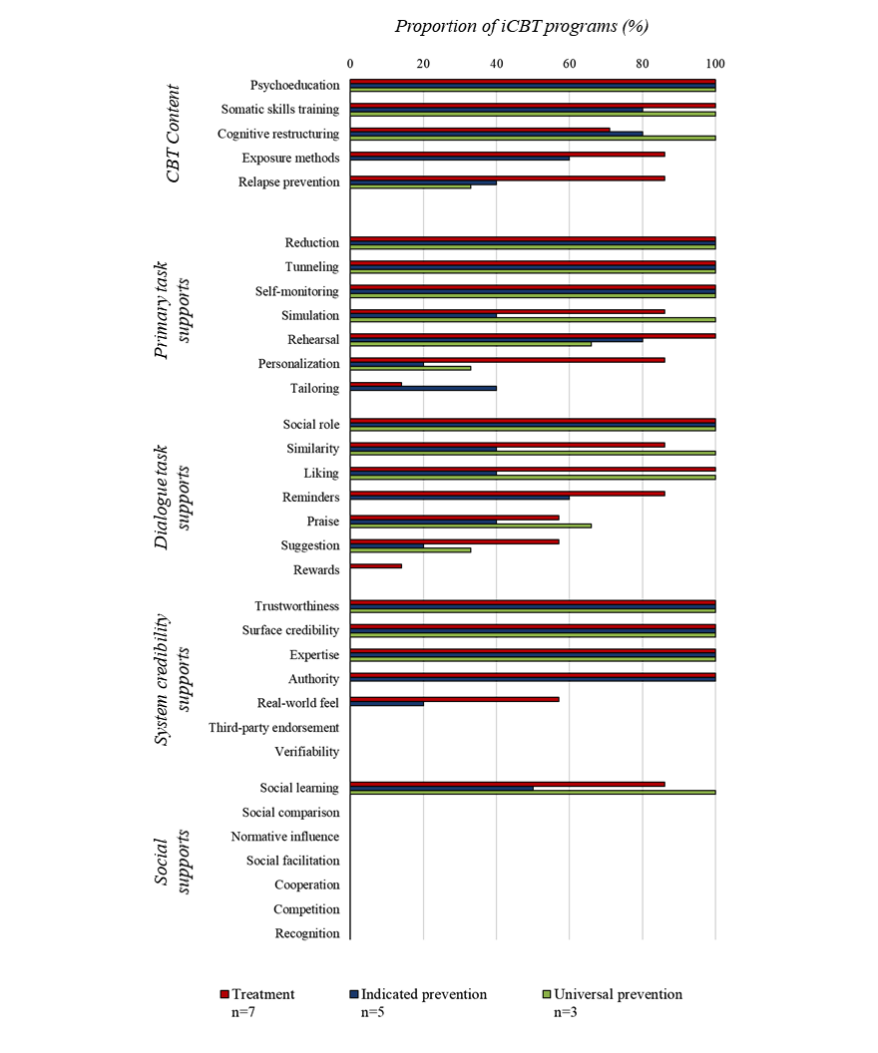
Frequency of the cognitive behavioral therapy (CBT) content and persuasive systems design features across 15 internet-based cognitive behavioral therapy (iCBT) programs, organized according to program type.

Figure 3 provides an overview of the proportion of iCBT programs that incorporated specific CBT content and PSD features, organized according to program type. All programs described themselves as *CBT-based* and contained AACAP recommended CBT components, although considerable variability in the type and quantity of components was found based on program type and age group of target users. Many programs also integrated techniques with an interpersonal focus, such as assertiveness training and problem-solving skills, to reduce environmental stressors and enhance social support [104]. Psychoeducation and somatic skills training were found in all iCBT programs. Cognitive restructuring was reported in more than half of the treatment programs and in nearly all the indicated and universal prevention programs. Relapse prevention was incorporated in a minority of prevention-based programs. Exposure methods were not delivered to users of universal prevention preventions.

Treatment programs incorporated the most PSD features, followed by indicated prevention and then universal prevention programs. Out of the 4 PSD support categories, features from the primary task support and dialogue support categories were most widely used. In terms of primary task supports, iCBT programs of all IOM program types incorporated reduction and tunneling to regulate the logical and incremental presentation of module content to users, mimicking the progressive delivery format of face-to-face CBT. Self-monitoring of users’ iCBT progress was also a primary task support feature common to all programs. Social role, a dialogue support feature, created a virtual presence of *others* in the program through Web- or email-based messaging between the user and therapist or recurring graphics or videos of real or animated peers. System credibility support features, trustworthiness, and surface credibility, although not explicitly reported, were inherent in all iCBT programs, as they were designed and delivered (tested) within a research study (ie, use of confidentiality and consent processes and declared academic affiliations). Authority features were associated with program content that was presented by a *reliable source* (ie, therapist) and was, therefore, only incorporated in treatment and universal prevention programs with adjunct therapist involvement. Social learning was the only social support feature included in iCBT programs, but not all indicated prevention programs utilized it. iCBT from different program types varied to the greatest extent on their use of personalization, tailoring, reminders, liking, and similarity features. Multimedia Appendix 1 provides additional examples of how PSD features were reported in the documents included in this synthesis.

#### Outcomes: Changes in Anxiety Post Intervention

Across program types, there was an overall trend for reduced anxiety symptoms among children and adolescents who received iCBT. An overview of the outcomes is provided in Multimedia Appendix 5. Among treatment programs, anxiety diagnoses, clinical severity, and parent- and user-reported symptoms were reduced post intervention in 98.5% of studies. Among indicated prevention programs, anxiety diagnoses, parent- and user-reported symptoms were reduced post intervention in 100% of studies. Among universal prevention programs, user-reported anxiety symptoms were reduced post intervention in 83.3% of studies.

### Key Relationships Between Internet-Based Cognitive Behavioral Therapy Program Contexts, Persuasive Systems Design Mechanisms, and Outcomes

We found that reductions in anxiety outcomes were reported across iCBT programs with many shared mechanisms and delivery contexts. Self-monitoring, simulation, social role, similarity, social learning, and rehearsal were common PSD features across all program types; however, mechanisms for customizing program content (ie, personalization and tailoring) distinguished treatment, indicated prevention, and universal prevention programs from one another. The key aspect of iCBT context that supported the mechanism-outcome interactions was adjunct support. The adjunct support person (eg, therapist, parent, and teacher), their expertise, and the intensity and frequency of their communication (eg, weekly personalized feedback and technical troubleshooting as needed) was associated with the program type, and, therefore, also the characteristics of users, such as age and symptom severity. In this way, treatment programs received the greatest amount of adjunct support relative to indicated and universal prevention programs.

### Context-Mechanism-Outcome Configurations

The refined set of context-mechanism-outcome configurations is summarized in Table 2, according to program type. Configurations, organized by PSD mechanism, are based on included information from documents that ranged in level of contribution (low to high).

**Table 2:** Summary of the 11 context-mechanism-outcome configurations for internet-based cognitive behavioral therapy programs for children and adolescents with anxiety.

Context—user characteristics and adjunct support			Mechanisms—PSD^a^ features and proposed function	Outcomes—trend in anxiety changes, pre- to postintervention^b^	Contributing programs	Mean MMAT^c^ score, %	Supporting studies where reductions of anxiety were found^d^, %
**Treatment programs: 1, 2, 3, 4, 5, 6, 7**							
	**Context: users were children diagnosed with an anxiety disorder(s); adjunct support provided by a therapist, parent and/or professional**						
		Configuration 1	Self-monitoring: to increase users’ attention to and comprehension of anxiety-related feelings or behaviors, track and present users’ program progress toward anxiety management or symptom reduction, and assess users’ accumulation of program-related knowledge	Reductions in user- and parent-reported symptoms, diagnoses, and clinical severity	Programs 1-7	88	98.5
		Configuration 2	Simulation + social role + similarity + social learning: to normalize users’ experience of anxiety and increase motivation or willingness to improve their mood and model the application of new anxiety management skills	Reductions in user- and parent-reported symptoms, diagnoses, and clinical severity	Programs 1-6	91	97.4
		Configuration 3	Rehearsal: to provide opportunities for developing fear tolerance, reduction, and/or extinction and reinforce the application of program concepts, behavioral anxiety management strategies, and problem-solving skills	Reductions in user- and parent-reported symptoms, diagnoses, and clinical severity	Programs 1-7	88	98.5
		Configuration 4	Personalization + social role + trustworthiness + expertise + authority: to provide customized feedback on user’s program activity to increase accurate comprehension and application of anxiety management concepts and skills	Reductions in user- and parent-reported symptoms, diagnoses, and clinical severity	Programs 1-6	91	98.5
**Indicated prevention programs: 8, 9, 10, 11, 12**							
	**Context: users were children or adolescents with mild-to-severe anxiety symptoms; adjunct support was provided by a therapist, parent and/or researcher**						
		Configuration 5	Self-monitoring: to increase users’ attention to and comprehension of anxiety-related feelings or behaviors, track program progress toward anxiety management/symptom reduction, and assess users’ accumulation of program-related knowledge	Reductions in user- and parent-reported symptoms and diagnoses	Programs 8-12	89	100
		Configuration 6	Simulation + social role + similarity + social learning: to normalize users’ experience of anxiety and increase motivation or willingness to improve their mood and model the application of new anxiety management skills	Reductions in user- and parent-reported symptoms and diagnoses	Programs 8 and 11	100	100
		Configuration 7	Rehearsal: to provide opportunities for developing fear tolerance, reduction, and/or extinction and reinforce the application of program concepts, cognitive and behavioral anxiety management strategies, and problem-solving skills	Reductions in user- and parent-reported symptoms and diagnoses	Programs 8-12	89	100
		Configuration 8	Tailoring: to adapt program content based on user’s demographic or mental health condition to improve the relevance for each user	Reductions in user-reported symptoms and diagnoses	Programs 11 and 12	100	100
**Universal prevention programs: 13, 14, 15**							
	**Context: users were adolescents who are not required to have any anxiety symptoms; adjunct support was teacher-facilitated program administration**						
		Configuration 9	Self-monitoring: to increase users’ attention to and comprehension of anxiety-related feelings or behaviors, track and present users’ program progress toward anxiety management or symptom reduction, and assess users’ accumulation of program-related knowledge	Reductions in user-reported symptoms	Programs 13-15	70	83.3
		Configuration 10	Simulation + social role + similarity + social learning: to normalize users’ experience of anxiety and increase motivation or willingness to improve their mood and model the application of new anxiety management skills	Reductions in user-reported symptoms	Programs 13-15	70	83.3
		Configuration 11	Rehearsal: to provide opportunities for developing fear tolerance and reinforce the application of program concepts, cognitive and behavioral anxiety management strategies, and problem-solving skills	Reductions in user-reported symptoms	Programs 13 and 14	75	80

^a^PSD: persuasive systems design.

^b^Categorized according to type of anxiety measure used, although specific instruments varied among studies.

^c^MMAT: mixed methods appraisal tool.

^d^Percentage of studies reporting a reduction in anxiety for internet-based cognitive behavioral therapy participants from pre- to postintervention.

#### Treatment Programs, Configurations 1 to 4

##### Configuration 1: Self-Monitoring

Treatment programs for children with an anxiety disorder, delivered with adjunct therapist, parent, or professional support and include self-monitoring, may produce postintervention reductions in user’s anxiety (diagnoses, clinical severity, self-reported, and parent-reported symptoms). Self-monitoring was part of the workflow for each module of the BRAVE-Online program and included regular tracking of symptoms and interactive activities and end-of-module quizzes to “facilitate attention and comprehension of material” [36]. Chilled Out program participants were presented with a weekly progress chart based on their reports of anxiety interference in their daily lives [83]. During program tasks, self-monitoring was employed using automated *pop-ups* stating the accuracy of users’ entries (ie, corrective comments) to ensure understanding of important concepts [36]. The adjunct support therapist encouraged users to self-monitor and record details of their in vivo (real world or offline) practice activities [39,41], including changes in anxiety following exposure exercises [37].

##### Configuration 2: Simulation, Social Role, Similarity, and Social Learning

Treatment programs for children with an anxiety disorder, delivered with adjunct therapist, parent, or professional support and include simulation with a social role, similarity, and social learning features, may produce postintervention reductions in user’s anxiety (diagnoses, clinical severity, self-reported, and parent-reported symptoms). These features were evident in videos or animations of peers, cartoon, and real-life characters to illustrate the experience of different emotions and the application of therapeutic skills, such as goal setting, developing fear hierarchies, and completing exposure activities [36,37,41,83]. Age-appropriate characters “provided ‘models’ for the use of coping strategies to overcome anxiety problems” [36]. Role models were designed to be appealing and relatable to users and their anxiety-related challenges; they represented someone with whom “the child can identify [with] and will be more likely to learn from” [58] (similarity). In another treatment program [37], the development of exposure-based film scenes used for fear extinction [105,106] of dental procedures were based on the principles of observational learning and the development of self-efficacy [107]. Email communication between the user and adjunct therapist (social role) could mimic or complement simulations, as therapists provided additional anxiety management instructions, tutorials or helped users problem-solve and plan exposure activities related to their specific situation or fears [76].

##### Configuration 3: Rehearsal

Treatment programs for children with an anxiety disorder, delivered with adjunct therapist, parent, or professional support and include rehearsal features, may produce postintervention reductions in user’s anxiety (diagnoses, clinical severity, self-reported, and parent-reported symptoms). Rehearsal was incorporated in brief, interactive tasks to be completed during the module (eg, drag this sentence to the correct term and drop it there) [41], quizzes for comprehension (eg, recap or summary quizzes) [58], or more in-depth, application-based worksheets at the end of the module [37,90]. For example, in BRAVE-Online, “Participants consolidate[d] learning of these [anxiety management] strategies through completion of weekly [Web-based] homework tasks, known as ‘extreme challenges’” [36]. Postmodule rehearsal activities recommended users to apply their target skill in real life anxiety-provoking situations outside of the program (ie, exposure exercises) [58]. An adjunct therapist was available to help structure and monitor some of these rehearsal activities. For example, in preparation for exposure activities, a supportive telephone call or message from the therapist assisted the user in developing a suitable exposure hierarchy [36,88].

##### Configuration 4: Personalization, Social Role, Trustworthiness, Expertise, and Authority

Treatment programs for children with an anxiety disorder, delivered with adjunct therapist, parent, or professional support and include personalization, a social role, trustworthiness, expertise, and authority, may produce postintervention reductions in user’s anxiety (diagnoses, clinical severity, self-reported, and parent-reported symptoms). Personalization provided a sense of program relatedness or knowing of the user through automated or manual features based on demographic details or program activity of the user. For example, the user’s name and that of his or her adjunct therapist could be populated throughout the modules [42]. Personalized *pop-ups* with immediate and specific feedback (eg, explanations for correct and incorrect answers [36]) on quizzes and tasks were also provided. In addition, the adjunct therapist (social role) monitored users’ responses to tasks and homework assignments and provided personalized written feedback by email. Personalized feedback was used to “reinforce effort and success and provide corrective information if required” [36], to “answer questions and clarify treatment content, increase motivation and to help solve problems” [41], or to “[ensure] adolescents’ understanding of the program elements” [83]. As the therapist could access user-specific information stored within the program, a response could be crafted with objective and supportive input through the therapist’s professional lens (authority); therefore, trustworthiness and expertise were features considered to be inherent to this personalized feedback process.

#### Indicated Prevention Programs, Configurations 5 to 8

##### Configuration 5: Self-Monitoring

Indicated prevention programs for children or adolescents with mild-to-severe anxiety symptoms, delivered with adjunct therapist, parent, or researcher support and include self-monitoring, may produce postintervention reductions in user’s anxiety (diagnoses, self-reported, and parent-reported symptoms). Self-monitoring was incorporated in the Feeling Better program using standardized symptom assessments at the beginning of modules as a way “to monitor symptom change” [43] over the course of the program. For some programs, symptom tracking was an essential part of the ongoing risk management of users [32,43]. The program or the adjunct support therapist would respond (automatically or manually) to safety concerns that arose from these assessments by providing additional mental health coping resources. In addition to mood, the program tracked the user’s progress toward goal achievement via homework completion. Module quizzes in some programs [31,57] were a means for users to review his or her understanding of new program concepts or skills [57].

##### Configuration 6: Simulation, Social Role, Similarity, and Social Learning

Indicated prevention programs for children or adolescents with mild-to-severe anxiety symptoms, delivered with adjunct therapist, parent, or researcher support and include simulation with a social role, similarity, and social learning, may produce postintervention reductions in user’s anxiety (diagnoses, self-reported, and parent-reported symptoms). Simulation was incorporated in examples or demonstration videos of individuals (social role) “illustrat[ing] certain concepts in the program” [57], providing suggested solutions, or working through their problems (social learning) [43]. The examples and activities provided in the Feeling Better program were specific to target users and their reported *stressors* (similarity) and were employed to “encourage practice and enhance learning of material” [43].

##### Configuration 7: Rehearsal

Indicated prevention programs for children or adolescents with mild-to-severe anxiety symptoms, delivered with adjunct therapist, parent, or researcher support and include rehearsal, may produce postintervention reductions in user’s anxiety (diagnoses, self-reported, and parent-reported symptoms). The STAY COOL program described including evidence-based practice activities (rehearsal) for reducing physical and cognitive test anxiety symptoms and pairing these coping activities with desensitizing exposure tasks to improve the program’s effectiveness [31]. In the same program, postmodule quizzes presented users with “a less-threatening, relatively low stakes exposure by testing them on recently obtained information in an untimed scenario” [31]. In the internet-based cognitive behavioral skills program, *Talk Time* was used to prompt the mother (adjunct parental support) and child to discuss a therapy topic or work together on a task [57]. In addition, exposure hierarchies were used to guide users’ practice (rehearsal) outside of the program as well. Adjunct therapists could provide rehearsal support (eg, encouragement and suggestions), if necessary, through their communications with the user.

##### Configuration 8: Tailoring

Indicated prevention programs for children or adolescents with mild-to-severe anxiety symptoms, delivered with adjunct therapist, parent, or researcher support and include tailoring, may produce postintervention reductions in user’s anxiety (diagnoses, self-reported, and parent-reported symptoms). iCBT content was tailored according to the user’s symptom profile. In the Feeling Better program, “A standardized assessment of symptoms of distress… [was] built into the start and end of core program modules to monitor symptom change and to help the user choose customized streams of program content specific to their emotional distress [such as anxiety, depression, or stress]” [43]. Another program had gender-specific versions (male and female), so that therapeutic examples matched the sex of the user [43]. For the individually tailored iCBT program for adolescents, the adjunct therapist used results from a baseline diagnostic interview to select module content (ie, psychoeducation and case examples) that corresponded to the user’s primary anxiety concern [32]. According to Silfvernagel (2017), a tailored iCBT program was “designed to identify a participant’s unique symptom profile and to provide information and skills that are likely to be helpful based on said profile” [96], aiming to improve the usefulness of the intervention.

#### Universal Prevention Programs, Configurations 9 to 11

##### Configuration 9: Self-Monitoring

Universal prevention programs for adolescents with minimal or no symptoms, delivered with teacher facilitation and include self-monitoring, may produce postintervention reductions in user’s self-reported anxiety symptoms. The MoodGYM program provided anxiety and depression quizzes (self-monitoring) before and after each module. Adolescents’ answers to quizzes and other program tasks were saved in a *personal Web-based workbook* that could be accessed by them at any time [34], serving as a benchmark for which they could compare changes over the course of iCBT. Electronic questionnaires were administered to adolescent users of Thiswayup Schools who also received notification if their scores were above average [102]. In the case of all 3 universal prevention programs, a teacher was present for iCBT administration and could provide referral advice if an adolescent’s symptoms required professional follow-up [108].

##### Configuration 10: Simulation, Social Role, Similarity, and Social Learning

Universal prevention programs for adolescents with minimal or no symptoms, delivered with teacher facilitation, and include simulation with a social role, similarity, and social learning, may produce postintervention reductions in user’s self-reported anxiety symptoms. Cartoon vignettes (similarity and social role) provided examples of anxiety management behaviors and responses as a regular part of the modules (simulation and social learning). For example, at the beginning of the MoodGYM program, adolescent users were “introduced to six distinct characters that form the basis of examples and discussion. Each character has a specific way of dealing with stressful situations, which [were] explored in the program.” [34]. Similarly, Thiswayup Schools used a storyline of cartoon teenagers with anxiety or depression to demonstrate ways to solve real life problems [35].

##### Configuration 11: Rehearsal

Universal prevention programs for adolescents with minimal or no symptoms, delivered with teacher facilitation, that include rehearsal may produce postintervention reductions in user’s self-reported anxiety symptoms. The e-couch Anxiety and Worry program included rehearsal exercises *to help users understand themselves and others better* [99]. The MoodGYM program also provided opportunities for users to apply therapeutic strategies to their own situation. Both quizzes and homework exercises were incorporated for users to practice their skills. User’s answers were recorded in their Web-based workbook and could be accessed at any time [34]. Rehearsal activities appeared to focus on cognitive restructuring, problem-solving, and interpersonal skills.

## Discussion

### Principal Findings

Our study systematically documented important similarities and differences in the design and delivery of iCBT components across 15 existing programs, which to our knowledge, is the first study of its kind for children or adolescents with anxiety. Anxiety reductions were reported in more than 98% of studies we reviewed. Our use of realist synthesis methods enabled the development of 11 context-mechanism-outcomes configurations that hypothesized the PSD features (technology-based mechanisms) that might contribute to the observed reductions in anxiety symptoms (outcomes), as they relate to key user and delivery features (context). Our results point to the need for increased emphasis on PSD in the development, evaluation, and reporting of iCBT programs for children and adolescents with anxiety concerns and further research designed to establish their relationship with improved anxiety symptomatology.

### Design and Delivery Components of Internet-Based Cognitive Behavioral Therapy for Children and Adolescents With Anxiety

The 11 configurations included PSD features from all 4 support categories. However, some category features were more often linked to iCBT program effects than others. Our findings highlight the central role of primary task supports in iCBT interventions for children and adolescents with anxiety, followed by dialogue support and system credibility support categories. Only 1 social support feature was supported by our analysis. These findings are in line with others [29,109] who also found primary task supports to be the most frequently reported persuasive features in technology-based health interventions. As primary task support features are considered to “aid users in completing their tasks and tracking and achieving their goals” [110], they have a similar aim to the goal-directed nature of iCBT programs. Dialogue support features keep “the user active and motivated in using the system” [111], so the user has more time and opportunities to complete their intended behavior(s) in the program. Both primary task support and dialogue support features have been linked to intervention effectiveness in previous studies in other fields [112-114].

### Toward Explanatory Persuasive Systems Design—Informed Models of Internet-Based Cognitive Behavioral Therapy Effects With Children and Adolescents With Anxiety

All iCBT programs in this study contained multiple PSD features. Although detailing the frequency of PSD features in iCBT provides some insight into what a user does within a program (activity), this information does not describe the important patterns or combinations of PSD features or explain why a program may or may not be effective. However, our findings suggest that (1) no PSD feature is applied in isolation and will likely not *work* as such (ie, some synergy is expected and essential) and (2) different PSD features have different functions, so we cannot assume that more features are better. We identified features from within and across different PSD support categories that were employed simultaneously, suggesting some synergistic or additive effect in their grouping (eg, simulation—a primary task support + social role—a dialogue support + similarity—a dialogue support + social learning—a social support). There have been attempts to examine the quantity and combination of PSD features in relation to the effectiveness of other health-based programs [29,109,111,115-117], but the literature is inconsistent and inconclusive. Wildeboer et al [111] indicated a positive relationship between effect sizes and the number of PSD features used in an intervention [111]. Additive or synergistic effects between multiple features, such as simulation and rehearsal, have been reported [117]. In contrast, other features together may negate or interfere with their persuasive potential [117], depending on the features and what persuasive support category they are from [111]. Future studies are needed to systematically assess the use and combination of multiple features as they relate to program effects to optimize the design of programs.

Overlap with the proposed context-mechanism-outcome configurations we generated and the literature on internet-based interventions indicate larger patterns for how these features operate. For example, others have hypothesized that self-monitoring might be used to increase user’s knowledge, self-awareness, and ability to monitor and manage their health [118,119]. Simulation allows users to cognitively or physically play out hypothetical situations [120], such as health-related decision making [121], to observe their effects before applying strategies to the real world [122]. In face-to-face CBT studies, rehearsal promotes skill acquisition [123], self-efficacy, and confidence with health management techniques [124,125]; rehearsal has been critical to the cognitive improvements found during treatment [126-128]. We propose that rehearsal may have a similar function in iCBT to that in face-to-face CBT, justifying its use in iCBT programs.

The consistent incorporation of specific key PSD features (rehearsal, self-monitoring, and simulation) in configurations across all program types indicates that these may be *signature features* that appeared to be particularly effective at producing the desired effects of iCBT regardless of the program type—perhaps because of the mental (psychological) activity and the interactions (effortful, suggestive, and engaging) between the user, the program content, and its features they incite. Our proposed key PSD features may produce symptom reductions in iCBT because they initiate higher-order cognitive processes, such as information recall, mental reflexivity, and future planning, much similar to the CBT content in these programs as well (ie, cognitive restructuring) [129], that may lead to longer lasting changes in learning and behavior. This observation supports a foundational conceptualization of persuasive systems as being a medium or tool for behavior change [19,130].

Differences in key PSD features may distinguish iCBT programs of one program type from another (ie, indicated prevention programs used tailoring whereas treatment programs used personalization). It has been recognized that user characteristics (eg, symptom severity and motivation), the focus of a program (eg, technological or therapeutic elements incorporated and the *at risk* behaviors targeted), and expected benefits (eg, degree of reduction in anxiety) differ depending on program type (eg, universal prevention, indicated prevention, and treatment) [53]. It may be that as the risk level and severity of symptoms of users increases from universal prevention (general population) to indicated prevention (low to high risk) to treatment programs (a diagnosed disorder), so does the program’s ability to adapt to user characteristics to improve its applicability and potential effectiveness (eg, providing relevant content based on user’s age and providing individual feedback on user’s practice activities). We believe maintaining the program type categorization is important in future testing of the PSD-based hypotheses as this categorization may help account for the distinct design and delivery components and the measures of effectiveness used (eg, primary outcomes, instruments, and significance level) across program types. Taking the unique contexts, mechanisms, and outcomes across program types into consideration will also help prepare the evidence base for implementation efforts of iCBT for anxious children and adolescents, for example, identifying important aspects of delivery setting, program support, or intervention features that may influence program effects [76,131,132].

### The Context-Mechanism Relationship

Realist synthesis methods focus on uncovering both the mechanisms of a complex intervention and their relationship to context [17,133-135]. We observed the important effect that the delivery context had on the PSD mechanisms that were included in iCBT programs, further confirming the importance of examining iCBT programs of a similar *program type* together. For example, we found that the context of all universal prevention programs involved widespread delivery in schools, during regular class periods, to all students in attendance, by a teacher with no specialized mental health training. This aspect of context differed considerably from indicated prevention and treatment programs that had a primarily *self-led* delivery format (ie, users could log into the program from any location at any time) where minimal, but some, Web- or email-based interaction with an adjunct support person was provided. PSD features could be affected by program contexts in a way that determined their presence or absence and the quality or how they were delivered. An example of this is that personalized feedback was provided to users only if an adjunct therapist was available to craft and deliver the message—a feature provided to users of treatment and indicated prevention programs. Another example is, in terms of iCBT practice, at-home or clinic-based delivery of programs required users to complete Web-based homework (rehearsal), whereas with some school-based universal prevention programs, paper-based homework (non-Web-based) was assigned to users during class.

School-based universal prevention programs have aspects of context (ie, setting of program use) that are different from indicated prevention and treatment programs, making their design and delivery unique [136]. In this study, we did not include the setting (home, clinic, or school delivery) in the proposed configurations as an understanding of how this context relates to specific PSD features to affect users’ anxiety did not emerge in our analyses. However, indications of a relationship between use setting, adjunct support, and program type (eg, the self-led delivery format of treatment and indicated prevention programs) was evident, indicating that the consideration of the impact of setting in future studies is warranted.

### Strengths and Limitations

We took a high-level perspective to develop hypotheses that may explain the effects of iCBT as a complex intervention. To our knowledge, this is the first study to systematically describe what and how PSD features may relate to symptom reductions in iCBT across programs for children and adolescents with anxiety. Although our findings may be similar to those of adult-based studies of internet-based interventions (eg, rehearsal [117] and self-monitoring [118]) or intuitive to researchers and developers in the iCBT field, no formal exploration of the effects of the PSD features on iCBT program outcomes for children and adolescents has previously been conducted. Our study acknowledged that there might be PSD features unique to programs designed for users within our age range (eg, social learning and peer demonstrations, ie, simulation, seemed especially important for children and adolescents using iCBT). Previous research suggests that the age and developmental stage of program users (eg, cognitive development: autonomous thinking and socioemotional: theory of mind) can affect the acceptability of an intervention [16,137,138] and intervention features (eg, tailoring, interactivity, and reinforcement) [139-141], indicating that there are unique iCBT design and delivery considerations to account for with children and adolescents that cannot be presupposed based on the adult literature [142,143].

This review has several strengths. We followed established and rigorous methods for conducting and reporting realist syntheses [24,26,27]. We included diverse, high-quality evidence from published and gray literature and used mixed methods for our analysis. Our approach to program evaluation was *inward-looking* in that it used data from iCBT programs and its users only. This allowed us to focus our analytic efforts to uncovering the within-iCBT relationships between design and delivery features (program contexts and mechanisms) that may produce the outcomes observed. With increasing emphasis being placed on the need for theory to guide internet-based intervention development [144-146], especially theories that consider intervention content, technology, and context together [147], our findings may inform theoretical developments in the field by providing new ideas around intervention processes and elements to test in future clinical trials of program effectiveness. We took some important first steps in the theory-building process (laying a foundation of evidence) by bringing together the fragmented and diverse data of iCBT programs, attempting to clearly define and report iCBT design and delivery features, highlighting important relationships between variables [24,148], and creating generalizable hypotheses. Another strength of this study is our use of the PSD and IOM models to organize the collection, analysis, interpretation, and presentation of data [149]. Although not applied by the original authors of the included documents, the models helped us identify and link contexts, mechanisms, and outcomes in a systematic way within and across programs.

Several challenges placed limitations on our findings. The 11 context-mechanism-outcome configurations we developed were dependent on the level of detail provided in the documents included in this synthesis. iCBT program descriptions were brief and details around therapeutic or technological features used (and associated theory or justification) were limited. Thus, the specifications of each technological (PSD) feature are not accounted for with our approach (which required a high level of abstraction) and readers should consider that the differences within features of the same type may be just as large as the differences across feature types (eg, rehearsal activities may differ among iCBT programs but these differences are not included in our configurations). We hope the hypotheses that we have generated can be applied to more detailed studies in the future that explore this important issue. In addition, few ineffective interventions (those that did not generate anxiety reductions) were identified for our review; therefore, we were unable to explore aspects of the delivery context or PSD features that might contribute to undesirable treatment effects with iCBT. As the dissemination and use of reporting standards (eg, Consolidated Standards of Reporting Trials–eHealth [150]) and requirements to document the design and delivery components for internet-based interventions become more common, we may not need to rely on additional models to operationalize data for comparisons across studies. We also acknowledge that information on other factors (ie, mediators or moderators) that may affect how iCBT programs work, such as user’s psychological characteristics (eg, cognitive processing style, beliefs or attitudes, skills, and literacy [151]), user engagement (eg, adherence, satisfaction, and motivation [152]), or environmental and cultural influences (eg, health care policies, user’s location, and societal perceptions of health), were not included in the configurations because of the lack of or inconsistent reporting. Thus, our proposed hypotheses for how iCBT programs for children and adolescents with anxiety work provide a constructive start to understanding their function but may not be complete. For example, once we better understand user characteristics, we may be able to identify subgroups of users who respond to iCBT, or some features of it, more than others. Although organizing our findings by program types led to a redundancy in the PSD mechanisms in configurations across program types, this redundancy also demonstrated the similarities that were universally found in iCBT programs. In the future, we recommend a more *formal* consideration of program type (ie, explicitly identifying the program’s targeted level of prevention) before designing and evaluating a program, as there are important differences in the target users, program design and delivery, and outcome measures used that may have a significant impact on program effects that should not be overlooked.

### Future Directions

As more complex and sophisticated technological mediums or delivery methods (ie, mobile phones and wearables) and features (ie, gamification, virtual reality, and virtual agents) are being developed and incorporated into new technology-based treatments, we need to understand the *first principles* for how the individual and most basic applications of PSD features in iCBT programs work, so we can *scale up* our understanding of their effects in parallel with advancing technology and the complexity of program design. This review highlights 2 recommendations for future directions in the iCBT field.

First, studies designed to assess the impact and functions of identified program components and identify other components that are relevant for the design of new iCBT programs for children and adolescents with anxiety are needed. Evaluating individual program features to understand their theoretical level of action (what the feature intends to do; eg, based on CBT or the PSD model), instantiation (how the feature was executed; eg, timing and volume), quality (a distinguishing aspect of the feature; eg, degree of personalization, size, or color), and their effect (the result or consequence of a feature; eg, initiating and reinforcing behavior) [153-156] may provide insights into what the most persuasive features are and how they can be deliberately combined to support users’ desired behavior change.

Evaluation of individual features requires the use of certain methodological frameworks [157,158] and study designs that allow for more timely feedback, iteration, and fewer resources for testing (ie, participants, multiple health care centers, and funds). For example, modeling and predictor analyses [159,160], multifactorial designs [161-163], trials with multiple treatment arms [164], adaptive evaluation strategies (ie, the multiphase optimization strategy [165,166]), or the use of mixed methods and the triangulation of data [167,168] may be attractive alternatives to traditional clinical trials [169].

Second, to advance our understanding of the causal mechanisms that underpin effective iCBT programs, we will need to address what and how therapeutic content (ie, CBT skills) is delivered using PSD (technology-based) features to produce the intended and actual attitude or behavior changes. This will involve developing a framework that integrates the PSD model with the CBT framework and a theory of behavior change (for a review of theories see [170]) to identify specific combinations of therapeutic content and technological features designed to help users meet their health goals. A holistic framework by Wang et al [171] combines behavioral theories and the PSD model to provide a starting point for more theoretical and comprehensive designing, reporting, and evaluation of persuasive systems [171].

### Conclusions

Although iCBT effectiveness for children and adolescents with anxiety has been demonstrated, not all programs benefit users in the same way. This leaves room for programs to be further optimized. PSD (technological) features can be intentionally selected and incorporated into the design and delivery of iCBT programs, making it an aspect of treatment that is under the control of developers. The hypotheses that we generated suggest that multiple key PSD features may work together to help users actively engage with therapeutic content and practice newly acquired skills. The type and degree of adjunct support will vary based on the level of prevention and user characteristics (ie, symptom severity) the program was designed to target and can influence what and how certain features operate within the program. The key PSD features and aspects of context identified require formal testing to understand whether, and to what extent, they are effective and how they function. These next steps may involve new conceptualizations of effectiveness and evaluation methods. As we improve our understanding of how the components of iCBT work (their proposed purpose) and what users prefer and need, we can create programs with better objective and subjective effectiveness. This systematic and deliberate approach to iCBT design and evaluation will increase the pool of evidence-based interventions available to prevent and treat anxiety in children and adolescents.

## Appendix

Multimedia Appendix 1 The persuasive systems design (PSD) model.

Multimedia Appendix 2 The candidate Context-Mechanism-Outcome configurations.

Multimedia Appendix 3 Document electronic search strategy.

Multimedia Appendix 4 The level of contribution and methodological quality of documents included in the synthesis.

Multimedia Appendix 5 Overview of the preintervention to postintervention changes in anxiety (outcomes) based on the total number of measures, studies, and iCBT programs across program types.

## Abbreviations

AACAP: American Academy of Child and Adolescent Psychiatry
CBT: cognitive behavioral therapy
CIHR: Canadian Institutes of Health Research
eHealth: electronic health
iCBT: internet-based cognitive behavioral therapy
IOM: Institute of Medicine
MMAT: Mixed Methods Appraisal Tool
PSD: persuasive systems design

## References

[1] Beesdo K, Knappe S, Pine DS (2009). Anxiety and anxiety disorders in children and adolescents: developmental issues and implications for DSM-V. Psychiatr Clin North Am.

[2] Costello EJ, Egger HL, Angold A, Ollendick TH, March JS (2004). A developmental epidemiology of anxiety disorders. Phobic and Anxiety Disorders in Children and Adolescents: A Clinician's Guide to Effective Psychosocial and Pharmacological Interventions.

[3] Creswell C, Waite P, Cooper PJ (2014). Assessment and management of anxiety disorders in children and adolescents. Arch Dis Child.

[4] Arnberg FK, Linton SJ, Hultcrantz M, Heintz E, Jonsson U (2014). Internet-delivered psychological treatments for mood and anxiety disorders: a systematic review of their efficacy, safety, and cost-effectiveness. PLoS One.

[5] Compton SN, March JS, Brent DA, Albano AM, Weersing VR, Curry J (2004). Cognitive-behavioral psychotherapy for anxiety and depressive disorders in children and adolescents: an evidence-based medicine review. J Am Acad Child Adolesc Psychiatry.

[6] Connolly SD, Bernstein GA, Work Group on Quality Issues (2007). Practice parameter for the assessment and treatment of children and adolescents with anxiety disorders. J Am Acad Child Adolesc Psychiatry.

[7] Seligman LD, Ollendick TH (2011). Cognitive-behavioral therapy for anxiety disorders in youth. Child Adolesc Psychiatr Clin N Am.

[8] Grist R, Croker A, Denne M, Stallard P (2019). Technology delivered interventions for depression and anxiety in children and adolescents: a systematic review and meta-analysis. Clin Child Fam Psychol Rev.

[9] Orlowski S, Lawn S, Antezana G, Venning A, Winsall M, Bidargaddi N, Matthews B (2016). A rural youth consumer perspective of technology to enhance face-to-face mental health services. J Child Fam Stud.

[10] Ye X, Bapuji SB, Winters SE, Struthers A, Raynard M, Metge C, Kreindler SA, Charette CJ, Lemaire JA, Synyshyn M, Sutherland K (2014). Effectiveness of internet-based interventions for children, youth, and young adults with anxiety and/or depression: a systematic review and meta-analysis. BMC Health Serv Res.

[11] Pennant ME, Loucas CE, Whittington C, Creswell C, Fonagy P, Fuggle P, Kelvin R, Naqvi S, Stockton S, Kendall T, Expert Advisory Group (2015). Computerised therapies for anxiety and depression in children and young people: a systematic review and meta-analysis. Behav Res Ther.

[12] Rooksby M, Elouafkaoui P, Humphris G, Clarkson J, Freeman R (2015). Internet-assisted delivery of cognitive behavioural therapy (CBT) for childhood anxiety: systematic review and meta-analysis. J Anxiety Disord.

[13] Ebert DD, Zarski AC, Christensen H, Stikkelbroek Y, Cuijpers P, Berking M, Riper H (2015). Internet and computer-based cognitive behavioral therapy for anxiety and depression in youth: a meta-analysis of randomized controlled outcome trials. PLoS One.

[14] Podina IR, Mogoase C, David D, Szentagotai A, Dobrean A (2015). A meta-analysis on the efficacy of technology mediated CBT for anxious children and adolescents. J Rat-Emo Cognitive-Behav Ther.

[15] Vigerland S, Lenhard F, Bonnert M, Lalouni M, Hedman E, Ahlen J, Olén O, Serlachius E, Ljótsson B (2016). Internet-delivered cognitive behavior therapy for children and adolescents: a systematic review and meta-analysis. Clin Psychol Rev.

[16] Hollis C, Falconer CJ, Martin JL, Whittington C, Stockton S, Glazebrook C, Davies EB (2017). Annual research review: digital health interventions for children and young people with mental health problems - a systematic and meta-review. J Child Psychol Psychiatry.

[17] Craig P, Dieppe P, Macintyre S, Michie S, Nazareth I, Petticrew M, Medical Research Council Guidance (2008). Developing and evaluating complex interventions: the new Medical Research Council guidance. Br Med J.

[18] Chatterjee S, Price A (2009). Healthy living with persuasive technologies: framework, issues, and challenges. J Am Med Inform Assoc.

[19] Fogg BJ (2002). Persuasive Technology: Using Computers to Change What We Think and Do.

[20] Oinas-Kukkonen H, Harjumaa M (2009). Persuasive systems design: key issues, process model, and system features. Commun Assoc Inf Syst.

[21] Calear AL, Batterham PJ, Poyser CT, Mackinnon AJ, Griffiths KM, Christensen H (2016). Cluster randomised controlled trial of the e-couch anxiety and worry program in schools. J Affect Disord.

[22] Spence SH, Donovan CL, March S, Kenardy JA, Hearn CS (2017). Generic versus disorder specific cognitive behavior therapy for social anxiety disorder in youth: a randomized controlled trial using internet delivery. Behav Res Ther.

[23] Dickson M, Riddell H, Gilmour F, McCormack B (2017). Delivering dignified care: a realist synthesis of evidence that promotes effective listening to and learning from older people's feedback in acute care settings. J Clin Nurs.

[24] Pawson R, Greenhalgh T, Harvey G, Walshe K (2004). Semantic Scholar.

[25] Pawson R, Greenhalgh T, Harvey G, Walshe K (2005). Realist review--a new method of systematic review designed for complex policy interventions. J Health Serv Res Policy.

[26] Pawson R, Tilley N (1997). Realistic Evaluation.

[27] Wong G, Westhorp G, Manzano A, Greenhalgh J, Jagosh J, Greenhalgh T (2016). RAMESES II reporting standards for realist evaluations. BMC Med.

[28] Oinas-Kukkonen H, Harjumaa M (2008). A Systematic Framework for Designing and Evaluating Persuasive Systems. Proceedings of the International Conference on Persuasive Technology.

[29] Lehto T, Oinas-Kukkonen H (2011). Persuasive features in web-based alcohol and smoking interventions: a systematic review of the literature. J Med Internet Res.

[30] Calear AL, Christensen H, Mackinnon A, Griffiths KM (2013). Adherence to the MoodGYM program: outcomes and predictors for an adolescent school-based population. J Affect Disord.

[31] Baker RW (2010). Repository Home.

[32] Silfvernagel K, Gren-Landell M, Emanuelsson M, Carlbring P, Andersson G (2015). Individually tailored internet-based cognitive behavior therapy for adolescents with anxiety disorders: a pilot effectiveness study. Internet Interv.

[33] Spence SH, Donovan CL, March S, Gamble A, Anderson RE, Prosser S, Kenardy J (2011). A randomized controlled trial of online versus clinic-based CBT for adolescent anxiety. J Consult Clin Psychol.

[34] Calear AL, Christensen H, Mackinnon A, Griffiths KM, O'Kearney R (2009). The YouthMood Project: a cluster randomized controlled trial of an online cognitive behavioral program with adolescents. J Consult Clin Psychol.

[35] Wong N, Kady L, Mewton L, Sunderland M, Andrews G (2014). Preventing anxiety and depression in adolescents: a randomised controlled trial of two school based internet-delivered cognitive behavioural therapy programmes. Internet Interv.

[36] Spence SH, Donovan CL, March S, Gamble A, Anderson R, Prosser S, Kercher A, Kenardy J (2008). Online CBT in the treatment of child and adolescent anxiety disorders: issues in the development of brave–online and two case illustrations. Behav Cogn Psychother.

[37] Shahnavaz S (2016). Karolinska Institutet.

[38] March S, Spence SH, Donovan CL (2009). The efficacy of an internet-based cognitive-behavioral therapy intervention for child anxiety disorders. J Pediatr Psychol.

[39] Vigerland S, Thulin U, Ljótsson B, Svirsky L, Ost L, Lindefors N, Andersson G, Serlachius E (2013). Internet-delivered CBT for children with specific phobia: a pilot study. Cogn Behav Ther.

[40] Calear AL, Christensen H, Brewer J, Mackinnon A, Griffiths KM (2016). A pilot randomized controlled trial of the e-couch anxiety and worry program in schools. Internet Interv.

[41] Vigerland S, Ljótsson B, Thulin U, Öst LG, Andersson G, Serlachius E (2016). Internet-delivered cognitive behavioural therapy for children with anxiety disorders: a randomised controlled trial. Behav Res Ther.

[42] Spence SH, Holmes JM, March S, Lipp OV (2006). The feasibility and outcome of clinic plus internet delivery of cognitive-behavior therapy for childhood anxiety. J Consult Clin Psychol.

[43] Currie SL, McGrath PJ, Day V (2010). Development and usability of an online CBT program for symptoms of moderate depression, anxiety, and stress in post-secondary students. Comput Human Behav.

[44] American Psychiatric Association (2013). Diagnostic and Statistical Manual of Mental Disorders: DSM-5. Fifth Edition.

[45] McHugh ML (2012). Interrater reliability: the kappa statistic. Biochem Med (Zagreb).

[46] Pluye P, Robert E, Cargo M, Bartlett G (2011). http://mixedmethodsappraisaltoolpublic.pbworks.com/w/file/84371689/MMAT.pdf.

[47] Pluye P, Gagnon MP, Griffiths F, Johnson-Lafleur J (2009). A scoring system for appraising mixed methods research, and concomitantly appraising qualitative, quantitative and mixed methods primary studies in mixed studies reviews. Int J Nurs Stud.

[48] Pace R, Pluye P, Bartlett G, Macaulay AC, Salsberg J, Jagosh J, Seller R (2012). Testing the reliability and efficiency of the pilot mixed methods appraisal tool (MMAT) for systematic mixed studies review. Int J Nurs Stud.

[49] Mrazek PJ, Haggerty RJ, Institute of Medicine, Committee on Prevention of Mental Disorders (1994). Reducing Risks for Mental Disorders: Frontiers for Preventive Intervention Research.

[50] Kelders SM, Kok RN, Ossebaard HC, van Gemert-Pijnen JE (2012). Persuasive system design does matter: a systematic review of adherence to web-based interventions. J Med Internet Res.

[51] Markowitz JC, Weissman MM (2004). Interpersonal psychotherapy: principles and applications. World Psychiatry.

[52] Mufson LH, Moreau D, Weissman MM, Dorta KP (2011). Interpersonal Psychotherapy for Depressed Adolescents.

[53] Springer JF, Phillips J (2007). Safe and Drug-Free Schools and Communities Technical Assistance.

[54] Atkins S, Lewin S, Smith H, Engel M, Fretheim A, Volmink J (2008). Conducting a meta-ethnography of qualitative literature: lessons learnt. BMC Med Res Methodol.

[55] Noblit GW, Hare RD (1988). Meta-Ethnography: Synthesizing Qualitative Studies.

[56] Conaughton RJ, Donovan CL, March S (2017). Efficacy of an internet-based CBT program for children with comorbid high functioning autism spectrum disorder and anxiety: a randomised controlled trial. J Affect Disord.

[57] Keller ML (2009). An Internet Cognitive-Behavioral Skills-Based Program for Child Anxiety.

[58] March S (2008). The University of Queensland's Institutional Repository.

[59] Stasiak K, Merry SN, Frampton C, Moor S (2018). Delivering solid treatments on shaky ground: feasibility study of an online therapy for child anxiety in the aftermath of a natural disaster. Psychother Res.

[60] Vigerland S, Serlachius E, Thulin U, Andersson G, Larsson JO, Ljótsson B (2017). Long-term outcomes and predictors of internet-delivered cognitive behavioral therapy for childhood anxiety disorders. Behav Res Ther.

[61] Spence S (2011). Australian New Zealand Clinical Trials Registry (ANZCTR).

[62] Spence S (2011). Australian New Zealand Clinical Trials Registry (ANZCTR).

[63] Spence S (2011). Australian New Zealand Clinical Trials Registry (ANZCTR).

[64] Anderson RE, Spence SH, Donovan CL, March S, Prosser S, Kenardy J (2012). Working alliance in online cognitive behavior therapy for anxiety disorders in youth: comparison with clinic delivery and its role in predicting outcome. J Med Internet Res.

[65] Stasiak K (2012). Australian New Zealand Clinical Trials Registry (ANZCTR).

[66] Moor S (2012). Australian New Zealand Clinical Trials Registry (ANZCTR).

[67] March S, Spence SH, Donovan CL, Kenardy JA (2018). Large-scale dissemination of internet-based cognitive behavioral therapy for youth anxiety: feasibility and acceptability study. J Med Internet Res.

[68] Moor S, Williman J, Drummond S, Fulton C, Mayes W, Ward N, Dovenberg E, Whitaker C, Stasiak K (2019). ‘E’ therapy in the community: examination of the uptake and effectiveness of BRAVE (a self-help computer programme for anxiety in children and adolescents) in primary care. Internet Interv.

[69] Spence SH (2017). BRAVE-Online - Helping Young People Overcome Anxiety.

[70] Shahnavaz S (2015). ClinicalTrials.gov.

[71] Shahnavaz S, Hedman-Lagerlöf E, Hasselblad T, Reuterskiöld L, Kaldo V, Dahllöf G (2018). Internet-based cognitive behavioral therapy for children and adolescents with dental anxiety: open trial. J Med Internet Res.

[72] Serlachius E (2012). ClinicalTrials.gov.

[73] (2013). BUP.

[74] Serlachius E (2014). ClinicalTrials.gov.

[75] Serlachius E (2015). ClinicalTrials.gov.

[76] Vigerland S (2015). Open Archive - Karolinska Institutet.

[77] Vigerland S (2016). ClinicalTrials.gov.

[78] Jolstedt M, Wahlund T, Lenhard F, Ljótsson B, Mataix-Cols D, Nord M, Öst L, Högström J, Serlachius E, Vigerland S (2018). Efficacy and cost-effectiveness of therapist-guided internet cognitive behavioural therapy for paediatric anxiety disorders: a single-centre, single-blind, randomised controlled trial. Lancet Child Adolesc Health.

[79] Stjerneklar S, Hougaard E, Thastum M (2014). PURE - Medarbejdere - Aarhus Universitet.

[80] University of Aarhus (2015). ClinicalTrials.gov.

[81] Nielsen A, Gaardsvig MM,  Stjerneklar S, Thastum M (2016). 8th World Congr Behav Cogn Ther.

[82] Stjerneklar S, Hougaard E, Thastum M (2017). PURE - Medarbejdere - Aarhus Universitet.

[83] Stjerneklar S, Hougaard E, Nielsen AD, Gaardsvig MM, Thastum M (2018). Internet-based cognitive behavioral therapy for adolescents with anxiety disorders: a feasibility study. Internet Interv.

[84] Stjerneklar S, Hougaard E, Thastum M (2019). Guided internet-based cognitive behavioral therapy for adolescent anxiety: predictors of treatment response. Internet Interv.

[85] (2019). Macquarie University.

[86] Hudson J, Rapee R, Brockveld K, McLellan L, Schneider S, Stow L, Rozario M, Wallace M, Wong A (2014). Macquarie University.

[87] Serlachius E (2015). ClinicalTrials.gov.

[88] Nordh M, Vigerland S, Öst LG, Ljótsson B, Mataix-Cols D, Serlachius E, Högström J (2017). Therapist-guided internet-delivered cognitive-behavioural therapy supplemented with group exposure sessions for adolescents with social anxiety disorder: a feasibility trial. BMJ Open.

[89] (2015). BUP.

[90] Karbasi A, Haratian A (2018). The efficacy of internet-based cognitive behavioral therapy on the anxiety disorders among adolescent girls. Adv Biomed Res.

[91] Vivyan C (2015). Get Self Help.

[92] Keller M (2009). Anxiety and Depression Research Center at UCLA.

[93] Keller M, Craske M (2009). Anxiety and Depression Research Center at UCLA.

[94] Ramdhani N, Widjaja JD, Rahmawati N (2015). Internet supported cognitive behavior therapy to help students with shy-socially isolated problems. Procedia Soc Behav Sci.

[95] Bradley KL, Robinson LM, Brannen CL (2012). Adolescent help-seeking for psychological distress, depression, and anxiety using an internet program. Int J Ment Health Promot.

[96] Silfvernagel K (2017). Individually Tailored Internet-Based Cognitive Behavioural Therapy for Adolescents, Young Adults and Older Adults With Anxiety.

[97] Calear A (2010). Australian New Zealand Clinical Trials Registry (ANZCTR).

[98] Calear AL, Christensen H, Griffiths KM, Mackinnon A (2013). The Y-Worri project: study protocol for a randomised controlled trial. Trials.

[99] Griffiths K, Tayler G, Christensen H e-Couch Self Help.

[100] Christensen H, Griffiths K moodgym - Online Self-Help for Depression and Anxiety.

[101] Andrews G (2012). Australian New Zealand Clinical Trials Registry (ANZCTR).

[102] Andrews G Improve Your Wellbeing | This Way Up.

[103] Calear AL, Batterham PJ, Griffiths KM, Christensen H (2017). Generalized anxiety disorder stigma in adolescents: personal and perceived stigma levels and predictors. Stigma and Health.

[104] Lipsitz JD, Markowitz JC (2013). Mechanisms of change in interpersonal therapy (IPT). Clin Psychol Rev.

[105] Melamed BG, Yurcheson R, Fleece EL, Hutcherson S, Hawes R (1978). Effects of film modeling on the reduction of anxiety-related behaviors in individuals varying in level of previous experience in the stress situation. J Consult Clin Psychol.

[106] Golkar A, Selbing I, Flygare O, Ohman A, Olsson A (2013). Other people as means to a safe end: vicarious extinction blocks the return of learned fear. Psychol Sci.

[107] Bandura A (1977). Self-efficacy: toward a unifying theory of behavioral change. Psychol Rev.

[108] Neil AL, Batterham P, Christensen H, Bennett K, Griffiths KM (2009). Predictors of adherence by adolescents to a cognitive behavior therapy website in school and community-based settings. J Med Internet Res.

[109] Lehto T, Oinas-Kukkonen H (2010). Persuasive Features in Six Weight Loss Websites: A Qualitative Evaluation. Proceedings of the International Conference on Persuasive Technology.

[110] Lehto T, Oinas-Kukkonen H (2014). Explaining and predicting perceived effectiveness and use continuance intention of a behaviour change support system for weight loss. Behav Inform Technol.

[111] Wildeboer G, Kelders SM, van Gemert-Pijnen JE (2016). The relationship between persuasive technology principles, adherence and effect of web-based interventions for mental health: a meta-analysis. Int J Med Inform.

[112] Langrial S, Lehto T, Oinas-Kukkonen H, Harjumaa M, Karppinen P (2012). Native Mobile Applications For Personal Wellbeing: A Persuasive Systems Design Evaluation. Proceedings of the 16th Pacific Asia Conference On Information Systems.

[113] Drozd F, Lehto T, Oinas-Kukkonen H (2012). Exploring Perceived Persuasiveness of a Behavior Change Support System: A Structural Model. Proceedings of the International Conference on Persuasive Technology.

[114] Lehto T, Oinas-Kukkonen H, Drozd F (2012). Factors Affecting Perceived Persuasiveness of a Behavior Change Support System. Proceedings of the Thirty Third International Conference on Information Systems.

[115] Wahle F, Bollhalder L, Kowatsch T, Fleisch E (2017). Toward the design of evidence-based mental health information systems for people with depression: a systematic literature review and meta-analysis. J Med Internet Res.

[116] Lentferink AJ, Oldenhuis HK, de Groot M, Polstra L, Velthuijsen H, van Gemert-Pijnen JE (2017). Key components in ehealth interventions combining self-tracking and persuasive ecoaching to promote a healthier lifestyle: a scoping review. J Med Internet Res.

[117] Räisänen T, Lehto T, Oinas-Kukkonen H (2010). Practical Findings from Applying the PSD Model for Evaluating Software Design Specifications. Proceedings of the International Conference on Persuasive Technology.

[118] Watkins JA, Goudge J, Gómez-Olivé FX, Huxley C, Dodd K, Griffiths F (2018). mHealth text and voice communication for monitoring people with chronic diseases in low-resource settings: a realist review. BMJ Glob Health.

[119] Burke LE, Wang J, Sevick MA (2011). Self-monitoring in weight loss: a systematic review of the literature. J Am Diet Assoc.

[120] Fogg BJ (1999). Persuasive technologie. Commun ACM.

[121] Lieberman DA (2001). Management of chronic pediatric diseases with interactive health games: theory and research findings. J Ambul Care Manage.

[122] Beard L, Wilson K, Morra D, Keelan J (2009). A survey of health-related activities on second life. J Med Internet Res.

[123] Luxton DD, McCann RA, Bush NE, Mishkind MC, Reger GM (2011). mHealth for mental health: integrating smartphone technology in behavioral healthcare. Prof Psychol: Res Pract.

[124] Kuonanoja L, Langrial S, Lappalainen R, Lappalainen PP, Oinas-Kukkonen H (2015). Treating depression with a behavior change support system without face-to-face therapy. Trans Human-Computer Interact.

[125] Peng W (2009). Design and evaluation of a computer game to promote a healthy diet for young adults. Health Commun.

[126] Langrial S, Oinas-Kukkonen H, Lappalainen P, Lappalainen R (2014). Influence of Persuasive Reminders and Virtual Rehearsal on Information Systems for Sleep Deprivation. Pacific Asia Conference on Information Systems.

[127] Thorpe GL, Hecker JE, Cavallaro LA, Kulberg GE (2009). Insight versus rehearsal in cognitive-behaviour therapy: a crossover study with sixteen phobics. Behav Cogn Psychother.

[128] Morgenstern J, Longabaugh R (2000). Cognitive-behavioral treatment for alcohol dependence: a review of evidence for its hypothesized mechanisms of action. Addiction.

[129] Dobson KS (2013). The science of CBT: toward a metacognitive model of change?. Behav Ther.

[130] Fogg BJ (2002). The functional triad computers in persuasive roles: overview. Persuasive Technology: Using Computers to Change What We Think and Do.

[131] Hadjistavropoulos HD, Nugent MM, Dirkse D, Pugh N (2017). Implementation of internet-delivered cognitive behavior therapy within community mental health clinics: a process evaluation using the consolidated framework for implementation research. BMC Psychiatry.

[132] Mol M, Dozeman E, van Schaik DJ, Vis CP, Riper H, Smit JH (2016). The therapist's role in the implementation of internet-based cognitive behavioural therapy for patients with depression: study protocol. BMC Psychiatry.

[133] Berwick DM (2008). The science of improvement. J Am Med Assoc.

[134] Dalkin S, Greenhalgh J, Jones D, Cunningham B, Lhussier M (2015). What's in a mechanism? Development of a key concept in realist evaluation. Implement Sci.

[135] Greenhalgh T, Humphrey C, Hughes J, Macfarlane F, Butler C, Pawson R (2009). How do you modernize a health service? A realist evaluation of whole-scale transformation in London. Milbank Q.

[136] Langley AK, Nadeem E, Kataoka SH, Stein BD, Jaycox LH (2010). Evidence-based mental health programs in schools: barriers and facilitators of successful implementation. School Ment Health.

[137] Sauter FM, Heyne D, Michiel Westenberg P (2009). Cognitive behavior therapy for anxious adolescents: developmental influences on treatment design and delivery. Clin Child Fam Psychol Rev.

[138] Beidas RS, Edmunds J, Ditty M, Watkins J, Walsh L, Marcus S, Kendall P (2014). Are inner context factors related to implementation outcomes in cognitive-behavioral therapy for youth anxiety?. Adm Policy Ment Health.

[139] Goh DH, Ang RP, Tan HC (2008). Strategies for designing effective psychotherapeutic gaming interventions for children and adolescents. Comput Human Behav.

[140] Orlowski SK, Lawn S, Venning A, Winsall M, Jones GM, Wyld K, Damarell RA, Antezana G, Schrader G, Smith D, Collin P, Bidargaddi N (2015). Participatory research as one piece of the puzzle: a systematic review of consumer involvement in design of technology-based youth mental health and well-being interventions. JMIR Hum Factors.

[141] Radomski AD, Wozney L, McGrath P, Huguet A, Hartling L, Dyson MP, Bennett K, Newton AS (2019). Design and delivery features that may improve the use of internet-based cognitive behavioral therapy for children and adolescents with anxiety: a realist literature synthesis with a persuasive systems design perspective. J Med Internet Res.

[142] Cavanagh K, Bennett-Levy J, Richards DA, Farrand P, Christensen H, Griffiths KM, Klein B, Lau MA, Proudfoot J, Ritterband L, White J, Williams C (2010). Turn on, tune in and (don't) drop out: Engagement, adherence, attrition, and alliance with internet-based interventions. Oxford Guide to Low Intensity CBT Interventions - Oxford Guides to Cognitive Behavioural Therapy.

[143] Yardley L, Spring B, Riper H, Morrison LG, Crane DH, Curtis K, Merchant GC, Naughton F, Blandford A (2016). Understanding and promoting effective engagement with digital behavior change interventions. Am J Prev Med.

[144] Lippke S, Ziegelmann JP (2008). Theory-based health behavior change: developing, testing, and applying theories for evidence-based interventions. Appl Psychol.

[145] Bartholomew Eldredge LK, Markham CM, Ruiter RA, Fernandez ME, Kok G, Parcel GS (2016). Planning Health Promotion Programs: An Intervention Mapping Approach.

[146] Michie S, Johnston M (2012). Theories and techniques of behaviour change: developing a cumulative science of behaviour change. Health Psychol Rev.

[147] Kelders SM, Oinas-Kukkonen H, Oörni A, van Gemert-Pijnen JE (2016). Health behavior change support systems as a research discipline: a viewpoint. Int J Med Inform.

[148] Byrne D (2013). Evaluating complex social interventions in a complex world. Evaluation.

[149] Abbott PA, Foster J, Marin HD, Dykes PC (2014). Complexity and the science of implementation in health IT--knowledge gaps and future visions. Int J Med Inform.

[150] Eysenbach G, CONSORT-EHEALTH Group (2011). Consort-eHealth: improving and standardizing evaluation reports of web-based and mobile health interventions. J Med Internet Res.

[151] Ritterband LM, Thorndike FP, Cox DJ, Kovatchev BP, Gonder-Frederick LA (2009). A behavior change model for internet interventions. Ann Behav Med.

[152] Barello S, Triberti S, Graffigna G, Libreri C, Serino S, Hibbard J, Riva G (2015). eHealth for patient engagement: a systematic review. Front Psychol.

[153] Anderson JK, Wallace LM (2015). Applying the behavioural intervention technologies model to the development of a smartphone application (app) supporting young peoples’ adherence to anaphylaxis action plan. BMJ Innov.

[154] Klasnja P, Consolvo S, Pratt W (2011). How to Evaluate Technologies for Health Behavior Change in HCI Research. Proceedings of the SIGCHI Conference on Human Factors in Computing Systems.

[155] Mohr DC, Schueller SM, Montague E, Burns MN, Rashidi P (2014). The behavioral intervention technology model: an integrated conceptual and technological framework for ehealth and mhealth interventions. J Med Internet Res.

[156] Mohr DC, Schueller SM, Riley WT, Brown CH, Cuijpers P, Duan N, Kwasny MJ, Stiles-Shields C, Cheung K (2015). Trials of intervention principles: evaluation methods for evolving behavioral intervention technologies. J Med Internet Res.

[157] Mohr DC, Cheung K, Schueller SM, Brown CH, Duan N (2013). Continuous evaluation of evolving behavioral intervention technologies. Am J Prev Med.

[158] van Gemert-Pijnen JE, Nijland N, van Limburg M, Ossebaard HC, Kelders SM, Eysenbach G, Seydel ER (2011). A holistic framework to improve the uptake and impact of ehealth technologies. J Med Internet Res.

[159] Kline RB (2016). Principles and Practice of Structural Equation Modeling.

[160] Greenland S, Brumback B (2002). An overview of relations among causal modelling methods. Int J Epidemiol.

[161] Collins LM, Dziak JJ, Li R (2009). Design of experiments with multiple independent variables: a resource management perspective on complete and reduced factorial designs. Psychol Methods.

[162] Collins LM, Dziak JJ, Kugler KC, Trail JB (2014). Factorial experiments: efficient tools for evaluation of intervention components. Am J Prev Med.

[163] Dziak JJ, Nahum-Shani I, Collins LM (2012). Multilevel factorial experiments for developing behavioral interventions: power, sample size, and resource considerations. Psychol Methods.

[164] Freidlin B, Korn EL, Gray R, Martin A (2008). Multi-arm clinical trials of new agents: some design considerations. Clin Cancer Res.

[165] Almirall D, Nahum-Shani I, Sherwood NE, Murphy SA (2014). Introduction to SMART designs for the development of adaptive interventions: with application to weight loss research. Transl Behav Med.

[166] Collins LM, Murphy SA, Strecher V (2007). The multiphase optimization strategy (MOST) and the sequential multiple assignment randomized trial (SMART): new methods for more potent ehealth interventions. Am J Prev Med.

[167] Creswell JW (2014). Research Design: Qualitative, Quantitative and Mixed Methods Approaches.

[168] Fielding NG (2012). Triangulation and mixed methods designs: data integration with new research technologies. J Mix Methods Res.

[169] Chow SC, Chang M (2008). Adaptive design methods in clinical trials - a review. Orphanet J Rare Dis.

[170] Davis R, Campbell R, Hildon Z, Hobbs L, Michie S (2015). Theories of behaviour and behaviour change across the social and behavioural sciences: a scoping review. Health Psychol Rev.

[171] Wang Y, Fadhil A, Lange J, Reiterer H (2019). Integrating taxonomies into theory-based digital health interventions for behavior change: a holistic framework. JMIR Res Protoc.

