# Supplementary material for: Potential Reduction of Symptoms With the Use of Persuasive Systems Design Features in Internet-Based Cognitive Behavioral Therapy Programs for Children and Adolescents With Anxiety: A Realist Synthesis
Source: JMIR Ment Health. 2019 Oct 23;6(10):e13807. doi: 10.2196/13807 (PMC7017649; doi:10.2196/13807)
Supplement: Multimedia Appendix 1 [file mental_v6i10e13807_app1.pdf]

Multimedia Appendix 1. The persuasive systems design (PSD) model.

PSD feature	Description	iCBT program example
**Primary Task Supports:** Assists the user in completing their target behaviour		
Reduction	Reduces complex behaviour into simple tasks	Individual sections of the program will be made available to participants each week to prevent participants completing the program prematurely [30]
Tunneling	Guides a user through a process or experience	Modules follow the same general format: instructional presentation, followed by new skill practice, then a final quiz [31]
Tailoring	Tailors the experience to the potential needs, interests, personality or use context	Five versions of the treatment program were created and each one was adapted, through psychoeducation and examples, to fit a specific anxiety disorder [32]
Personalization	Personalizes content	Therapist constructs a brief feedback e-mail to the user after each session [33]
Self-monitoring	Keeps track of the user's performance or status towards goal achievement	Mood quizzes are completed at the beginning of each module and at the end of the program [34]
Simulation	Provides simulations to enable the user to observe link between cause and effect	Users follow a cartoon based storyline of teenagers with anxiety or depression solve real life problems [35]
Rehearsal	Provides a way for the user to rehearse a skill or task	The program provides opportunities to apply strategies to own example; it also includes quizzes and homework exercises [34]
**Dialogue Supports:** Provides computer-human communication to guide user toward target behaviour		
Praise	Offers praise as a form of feedback	Upon completion of a session, users are sent an automatic email from their therapist congratulating them on finishing their session [36]
Rewards	Rewards target behaviour	Participants received a diploma at the end of the treatment [37]
Reminders	Reminds the user of their target behaviour	Automatic email reminders were sent when participants had not completed the next session within one week [38]
Suggestion	Offers fitting suggestions	If the user reports high symptoms, specific services are recommended by the program [34]
Similarity	Reminds the user of themselves in some meaningful way	The language was adapted to suit the age group [32]
Liking	Is visually attractive for the user	Eye-catching graphics, sounds, games, and quizzes are used to maintain the adolescents' level of interest [33]

Social role	Adopts a social role	Users could send written messages to their therapist at any time during treatment [39]
**System Credibility Supports:** System design features increase user's perceptions of credibility		
Trustworthiness	Provides information that is truthful, fair and unbiased	Evidence-based information and strategies provided [40]
Expertise	Provides information showing knowledge, experience, and competence	The role of the psychologist was to answer questions and clarify treatment content, increase motivation and to help solve problems if necessary [41]
Surface credibility	Has a competent look and feel	The program was visually appealing and interesting with minimal amount of text per page [36]
Real-world feel	Provides information of the actual people behind its content and services	A brief biography of the program therapist was provided to users [42]
Authority	Refers to people in the role of authority	Each module asks the user to practice the strategies presented and review progress with their coach [43]
3rd party endorsement	Provides endorsements from other sources	Logos of academic affiliations were located on the website [34]
Verifiability	Provides means to verify the accuracy of the program via outside sources	A dental professional offered training and exposure at the clinic alongside the therapist's web-based guidance [37]
**Social Supports:** Leverages the interactions and influence of others		
Social learning	Can use the system to observe others performing tasks or behaviour	"Brave Buddy" is a cartoon character that provides a "model" in the use of anxiety coping strategies [36]
Social comparison	Can use the system to compare their performance to performance of others	Users can see the average score of their peers on a mood questionnaire
Normative influence	Leverages normative influence or peer pressure	User testimonials provided on the program website
Social facilitation	User discerns via the system that others are performing the behaviour along with them	Avatars indicate users currently active in the program
Cooperation	Leverages drive to cooperate to complete a task or behaviour	Peer forum allows users to share coping strategies
Competition	Leverages drive to compete against others in completing a task or action	Users challenge an animated character in a quiz
Recognition	Offers public recognition for a user or group	Program completion 'status' is shared to social media
