# Supplementary material for: Potential Reduction of Symptoms With the Use of Persuasive Systems Design Features in Internet-Based Cognitive Behavioral Therapy Programs for Children and Adolescents With Anxiety: A Realist Synthesis
Source: JMIR Ment Health. 2019 Oct 23;6(10):e13807. doi: 10.2196/13807 (PMC7017649; doi:10.2196/13807)
Supplement: Multimedia Appendix 2 [file mental_v6i10e13807_app2.pdf]

Multimedia Appendix 2. The candidate Context-Mechanism-Outcome configurations.

User characteristics	Delivery Context	PSD Mechanism	Anxiety Outcome
Children and/or adolescents with mild-to-moderate anxiety symptoms or an anxiety disorder(s)	Adjunct therapist, parent and/or professional support	Tailoring	Reduction in symptoms
Children and/or adolescents with mild-to-moderate anxiety symptoms or an anxiety disorder(s)	Adjunct therapist, parent and/or professional support	Personalization	Reduction in symptoms
Children and/or adolescents with mild-to-moderate anxiety symptoms or an anxiety disorder(s)	Adjunct therapist, parent and/or professional support	Self-monitoring	Reduction in symptoms
Children and/or adolescents with mild-to-moderate anxiety symptoms or an anxiety disorder(s)	Adjunct therapist, parent and/or professional support	Simulation + Social learning	Reduction in symptoms
Children and/or adolescents with mild-to-moderate anxiety symptoms or an anxiety disorder(s)	Adjunct therapist, parent and/or professional support	Rehearsal	Reduction in symptoms
Children and/or adolescents with mild-to-moderate anxiety symptoms or an anxiety disorder(s)	Adjunct therapist, parent and/or professional support	Suggestions	Reduction in symptoms
Children and/or adolescents with mild-to-moderate anxiety symptoms or an anxiety disorder(s)	Adjunct therapist, parent and/or professional support	Similarity + Liking	Reduction in symptoms
Children and/or adolescents with mild-to-moderate anxiety symptoms or an anxiety disorder(s)	Adjunct therapist, parent and/or professional support	Social role + Expertise	Reduction in symptoms
