# Supplementary material for: Potential Reduction of Symptoms With the Use of Persuasive Systems Design Features in Internet-Based Cognitive Behavioral Therapy Programs for Children and Adolescents With Anxiety: A Realist Synthesis
Source: JMIR Ment Health. 2019 Oct 23;6(10):e13807. doi: 10.2196/13807 (PMC7017649; doi:10.2196/13807)
Supplement: Multimedia Appendix 4 [file mental_v6i10e13807_app4.pdf]

Multimedia Appendix 4. The level of contribution and methodological quality of documents included in the synthesis.

Document	Document type and study design	Relevance			Rigor
		Level of Contribution			MMAT Score
		Context	Mechanism	Outcome	
**Program 1: BRAVE-Online for children and adolescents**					
[42]	Published study; Quantitative randomized	High	Medium	Medium	100%
[36]	Published study; Quantitative descriptive	High	High	Medium	100%
	Published study; Quantitative descriptive	High	High	Medium	100%
[58]	Thesis; Quantitative randomized	High	Medium	Medium	100%
	Thesis; Quantitative randomized	High	High	High	100%
[38]	Published study; Quantitative randomized	High	Medium	Medium	100%
[33]	Published study; Quantitative randomized	High	Medium	Medium	100%
[61]	Registered protocol; Quantitative non-randomized	Medium	Low	N/A	N/A
[62]	Registered protocol; Quantitative randomized	Medium	Low	N/A	N/A
[63]	Registered protocol; Quantitative randomized	Medium	Low	N/A	N/A
[64]	Published study; Quantitative non-randomized	Medium	Medium	N/A	N/A
[65]	Registered protocol; Quantitative randomized	Low	Low	N/A	N/A
[66]	Registered protocol; Quantitative descriptive	Low	Low	N/A	N/A
[59]	Published study; Quantitative non-randomized	High	High	N/A	N/A
[22]	Published study; Quantitative randomized	High	High	High	50%
[56]	Published study; Quantitative randomized	High	High	High	75%
[67]	Published study; Quantitative descriptive	High	High	High	75%
[68]	Published study; Quantitative descriptive	Low	Medium	Medium	75%
[69]	Program website; N/A	Low	Low	N/A	N/A
**Program 2: iCBT for children with anxiety disorders for children and adolescents with dental anxiety**					
[70]	Registered protocol; Quantitative randomized	Medium	Low	N/A	N/A
[37]	Thesis; Quantitative non-randomized	Medium	High	High	75%
[71]	Published study; Quantitative descriptive	Medium	High	Medium	100%

Document	Document type and study design	Relevance			Rigor
		Level of Contribution			MMAT Score
		Context	Mechanism	Outcome	
**Program 3: Internet-delivered CBT for children with anxiety disorders**					
[72]	Registered protocol; Quantitative non-randomized	Low	Low	N/A	N/A
[73]	Program website; N/A	Low	Low	N/A	N/A
[74]	Registered protocol; Quantitative non-randomized	Low	Low	N/A	N/A
[75]	Registered protocol; Quantitative randomized	Low	Low	N/A	N/A
[76]	Thesis; Quantitative randomized	High	Medium	Medium	75%
	Thesis; Quantitative non-randomized	High	Medium	High	100%
[77]	Registered protocol; Quantitative non-randomized	Low	None	N/A	N/A
[41]	Published study; Quantitative randomized	High	Medium	Medium	75%
[60]	Published study; Quantitative non-randomized	High	High	High	100%
[78]	Published study; Quantitative randomized	Medium	Medium	High	100%
**Program 4: Internet-delivered CBT for children with specific phobia**					
[39]	Published study; Quantitative non-randomized	High	Medium	Medium	100%
**Program 5: The Chilled Out program**					
[79]	Conference poster; N/A	Low	Low	N/A	N/A
[80]	Registered protocol; Quantitative descriptive	Low	Low	N/A	N/A
[81]	Conference abstract; Quantitative descriptive	Low	Low	N/A	N/A
[82]	Conference poster; Quantitative descriptive	Low	Low	Low	100%
[83]	Published study; Mixed methods	Medium	Medium	High	100%
[84]	Published study; Quantitative descriptive	Low	Medium	N/A	N/A
[85]	Study flyer; N/A	Low	Low	N/A	N/A
[86]	Program website; N/A	Low	Low	N/A	N/A
**Program 6: Group therapy supported iCBT for adolescents with social anxiety disorder**					
[90]	Registered protocol; Quantitative descriptive	Low	Low	N/A	N/A
[91]	Published study; Quantitative descriptive	Medium	High	Medium	100%
[92]	Program website; N/A	Low	Low	N/A	N/A

Document	Document type and study design	Relevance			Rigor
		Level of Contribution			MMAT Score
		Context	Mechanism	Outcome	
**Program 7: iCBT for anxiety disorders among adolescent girls**					
[93]	Published study; Quantitative randomized	Low	Low	Medium	25%
[94]	Program website; N/A	Low	Low	N/A	N/A
**Program 8: Internet cognitive-behavioural skills-based program**					
[87]	Published study; Quantitative randomized	High	High	High	100%
[88]	Program website; N/A	Low	Low	N/A	N/A
[89]	Study flyer; N/A	Low	Low	N/A	N/A
**Program 9: Internet-supported brief CBT for shy-socially isolated problem**					
[95]	Published study; Quantitative non-randomized	Low	Low	Medium	50%
**Program 10: STAY COOL system for test anxiety**					
[31]	Thesis; Quantitative randomized	High	High	High	75%
**Program 11: Feeling Better**					
[43]	Published study; Qualitative	High	High	N/A	100%
[96]	Published study; Mixed methods	Medium	Low	Low	100%
**Program 12: Individually tailored iCBT for adolescents**					
[32]	Published study; Quantitative randomized	High	Medium	Medium	100%
[97]	Thesis; Quantitative randomized	High	Medium	Low	100%
**Program 13: The e-couch Anxiety and Worry Program**					
[98]	Registered protocol; Quantitative randomized	Medium	Low	N/A	N/A
[99]	Registered protocol; Quantitative randomized	High	High	N/A	N/A
[40]	Published study; Quantitative randomized	High	High	High	50%
[21]	Published study; Quantitative randomized	High	High	Medium	50%
[100]	Program website; N/A	Low	Low	N/A	N/A
**Program 14: MoodGYM**					
[34]	Published study; Quantitative randomized	High	Medium	Medium	100%
[30]	Published study; Quantitative randomized	Medium	High	High	100%
[101]	Program website; N/A	None	None	N/A	N/A

Document	Document type and study design	Relevance			Rigor
		Level of Contribution			MMAT Score
		Context	Mechanism	Outcome	
**Program 15: Thiswayup Schools for Anxiety and Depression prevention courses**					
[102]	Registered protocol; Quantitative randomized	Medium	Low	N/A	N/A
[35]	Published study: Quantitative randomized	Low	Low	Medium	50%
[103]	Program website; N/A	None	Low	N/A	N/A
