# Supplementary material for: Potential Reduction of Symptoms With the Use of Persuasive Systems Design Features in Internet-Based Cognitive Behavioral Therapy Programs for Children and Adolescents With Anxiety: A Realist Synthesis
Source: JMIR Ment Health. 2019 Oct 23;6(10):e13807. doi: 10.2196/13807 (PMC7017649; doi:10.2196/13807)
Supplement: Multimedia Appendix 5 [file mental_v6i10e13807_app5.pdf]

Multimedia Appendix 5. Overview of the pre-intervention to post-intervention changes in anxiety (Outcomes) based on the total number of measures, studies, and iCBT programs across program types.

**Change in anxiety, pre- to post-intervention**	**Anxiety measures**			
	**User-reported symptoms**	**Parent-reported symptoms**	**Diagnosis**	**Clinical severity**
**Treatment programs 1, 2, 3, 4, 5, 6, 7**				
Reductions in anxiety for iCBT participants	22 measures used across 19 studies of programs 1, 2, 3, 4, 5, 6, 7 [22,33,60,66,67,71,76,78,84,88,90, 36-39,41,42,56, 58]	17 measures used across 17 studies of programs 1, 2, 3, 4, 5, 6 [22,33,60,66,71, 76,78,84,88,36–39,41, 42,56,58]	20 measures used across 18 studies of programs 1, 2, 3, 4, 5, 6 [22,33,60,66,71,76, 78,82,83,88,36–39, 41,42,56,58]	16 measures used across 16 studies of programs 1, 3, 4, 6 [33,36,76,78,82,83,88,104,38,39,42,56,58,60,63,66]
Increases in anxiety for iCBT participants	1 measure used in 1 study of program 1 [36]	—	—	—
**Indicated prevention programs 8, 9, 10, 11, 12**				
Reductions in anxiety for iCBT participants	4 measures used across 4 studies of programs 9, 10, 11, 12 [31,32,95–97]	1 measure used in 1 study of program 8 [57]	2 measures used in 2 studies of programs 8, 12 [32,57,97]	—
**Universal prevention programs 13, 14, 15**				
Reductions in anxiety for iCBT participants	9 measures used across 5 studies of programs 13, 14, 15 [21,30,35,40]	—	—	—
Increases in anxiety for iCBT participants	1 measure used in 1 study of program 10 [21]	—	—	—

iCBT: Internet-based cognitive behavioural therapy

Note: Multiple outcome measures may have been used in a single study. Also, some subgroup analyses were conducted. Please refer to individual studies for the results of each outcome measure.

Note: If two studies shared a partial or full dataset, results were counted as a single study, but both documents were cited. If one document contained two separate studies, two studies were reported but one document was cited.
